# Long-term priors influence visual perception through recruitment of long-range feedback

**DOI:** 10.1038/s41467-021-26544-w

**Published:** 2021-11-01

**Authors:** Richard Hardstone, Michael Zhu, Adeen Flinker, Lucia Melloni, Sasha Devore, Daniel Friedman, Patricia Dugan, Werner K. Doyle, Orrin Devinsky, Biyu J. He

**Affiliations:** 1 Neuroscience Institute, New York University Grossman School of Medicine, New York, NY 10016 USA; 2 Department of Neurology, New York University Grossman School of Medicine, New York, NY 10016 USA; 3 Department of Neurosurgery, New York University Grossman School of Medicine, New York, NY 10016 USA; 4 Department of Neuroscience and Physiology, New York University Grossman School of Medicine, New York, NY 10016 USA; 5 Department of Radiology, New York University Grossman School of Medicine, New York, NY 10016 USA

**Keywords:** Perception, Dynamical systems, Object vision

## Abstract

Perception results from the interplay of sensory input and prior knowledge. Despite behavioral evidence that long-term priors powerfully shape perception, the neural mechanisms underlying these interactions remain poorly understood. We obtained direct cortical recordings in neurosurgical patients as they viewed ambiguous images that elicit constant perceptual switching. We observe top-down influences from the temporal to occipital cortex, during the preferred percept that is congruent with the long-term prior. By contrast, stronger feedforward drive is observed during the non-preferred percept, consistent with a prediction error signal. A computational model based on hierarchical predictive coding and attractor networks reproduces all key experimental findings. These results suggest a pattern of large-scale information flow change underlying long-term priors’ influence on perception and provide constraints on theories about long-term priors’ influence on perception.

## Abstract (web summary)

Priors learnt from lifetime experiences influence perception. The authors show that when perception is congruent with a long-term prior, there is increased top-down input in the ventral visual stream, whereas bottom-up input is enhanced when perception is incongruent with prior.

## Introduction

Perception is much more than what meets the eye. Incoming visual input is actively shaped by internal processes such as attention^1,2^, expectation^3,4^, and prior knowledge^4–9^. It is well known that priors learnt from lifetime experiences powerfully influence perception^10–13^. For instance, due to the lifelong ‘light-comes-from-above’ prior, we perceive shapes with shading at the top as concave^14–16^. These long-term priors (i.e., priors that are stably encoded in the brain, reflecting repeated past experiences, or genetic influences) are context-independent and apply to novel experiences^17^. Yet, the neural mechanisms underlying long-term priors’ influence on perception remain elusive.

Two conflicting theories about the neural machinery underlying long-term priors’ influence on perception have been proposed. According to one theory, context-independent long-term priors act predominantly in a bottom-up fashion^14,17,18^, implemented in the very machinery that processes sensory information. This proposal is supported by findings showing that there is an over-representation of neurons tuned to cardinal orientations and centrifugal motion directions in early visual areas^13,19^, suggesting that neuronal tuning in early sensory processing already reflects common regularities in the sensory environment. By contrast, an alternative theory suggests that prior knowledge, including those learnt from long-term experiences, resides in higher-order brain regions and acts on perception primarily through top-down feedback^20,21^. Yet, although existing evidence suggests that prior knowledge acquired from task-dependent cues can influence perception through top-down feedback from frontoparietal cortices^22–24^, no study to date has shown a similar top-down mechanism for the influence of prior knowledge learnt from long-term experiences.

**Fig. 1:**
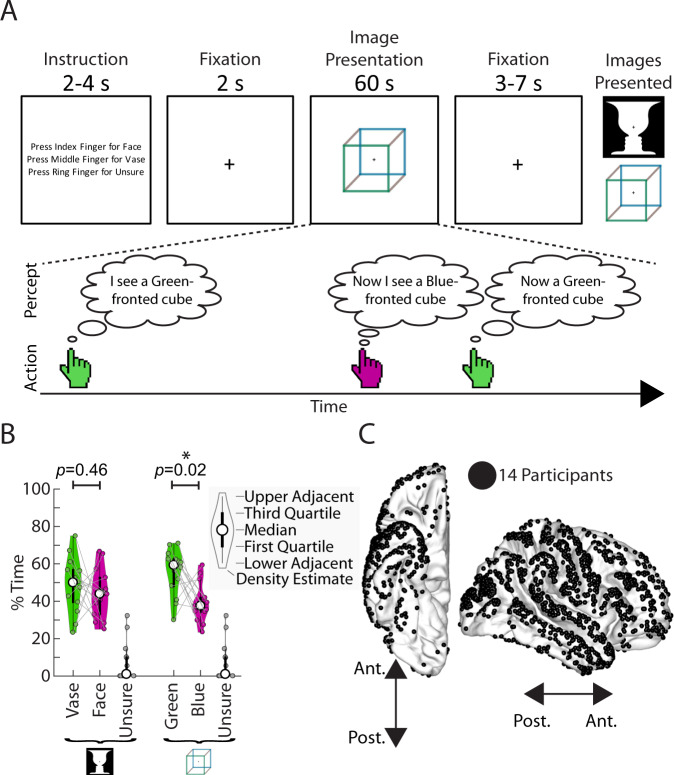
Paradigm, behavior, and electrode locations. **A** Task structure. Participants viewed ambiguous images presented for one minute at a time, and pressed buttons to indicate their alternating percepts. They were allowed to answer “unsure” for mixed percepts. The Necker cube and face-vase images were created by one of the authors (B.J.H.) and published in a previous study^33^. **B** Percentage of time spent in each of the possible percepts for the two images. Shown as a violin plot, which includes features of a boxplot (thick circle indicates median, thick black line is the inter-quartile range, and thin black line extend to the most extreme data points not considered outliers), as well as a density estimate of the distribution. Each thin circle represents one participant (*n* = 14). Significant difference in percentage time between the two percepts of an image was assessed with a two-sided Wilcoxon sign-rank test. **C** Electrode locations for all participants. Electrodes on the left hemisphere were mapped onto the right hemisphere for visualization purpose only. For electrode coverage in individual patients see Supplementary Fig. 1. Source data are provided as a Source Data file.

Ambiguous images offer a well-controlled experimental paradigm to address this question. When viewed, these images elicit constant switching of perceptual outcome between two plausible interpretations, such as the view-from-above and view-from-below perspectives of the Necker cube (Fig. 1A). Importantly, this perceptual switching is often asymmetrical, in a manner that reflects prior knowledge engrained from long-term experiences. For instance, the Necker cube is more often perceived as being viewed from above even though it is a symmetric figure, due to humans having viewed objects more often from above than from below throughout their lives—the so-called ‘view from above’ prior^25–27^. This phenomenon provides an ideal opportunity to examine how long-term priors guide perception and bias one perceptual outcome to be preferred despite symmetrical bottom-up evidence.

In line with the proposal postulating top-down influences of long-term priors, we hypothesized that when presented with ambiguous sensory input, prior knowledge learnt from lifetime experiences is recruited and fed back from higher-order brain areas to lower-order areas, manifesting as an increased feedback drive during the preferred (i.e., more commonly experienced) percept that is congruent with long-term prior. In addition, consistent with the predictive processing framework^28–30^, we hypothesized that during the non-preferred percept—the percept incongruent with long-term prior—there is a stronger prediction error signal manifesting as an increased feedforward drive in the same large-scale cortical network.

Despite decades of research on bistable perception^31,32^, few studies have probed the neural bases of perceptual asymmetry shaped by long-term priors. Moreover, the dynamic interactions between brain regions driving the ebb and flow of alternating percepts remain poorly understood, and previous studies using fMRI^33–35^ or magnetoencephalography^36^ to address this question suffer from poor temporal resolution or limitations in source localization, while recent studies^37,38^ using intracranial recordings have had a very small number of participants (*N* = 2) and only investigated the visual cortex.

To test our hypothesis, we presented two different ambiguous images (Necker cube and Rubin face-vase illusion; Fig. 1A) to patients undergoing invasive electrode monitoring for neurosurgical evaluation to treat pharmacologically resistant epilepsy. We collected the first extensive electrocorticography (ECoG) data set during bistable perception in 14 patients with 1321 analyzed electrodes covering all cortical lobes (Fig. 1C). With millisecond timing precision, accurate spatial localization, and widespread coverage, ECoG is ideal for probing dynamic information flow across large-scale brain networks. With this dataset, we investigated large-scale information flow during the preferred and non-preferred percept of ambiguous images, as well as neural activity underlying perceptual switching and the maintenance of a percept.

Here, we show that across both ambiguous images, the preferred percept is accompanied by enhanced top-down influences from the temporal to occipital cortex. By contrast, the non-preferred percept is accompanied by stronger feedforward activity in the same long-distance pathways. A computational model incorporating attractor-network and hierarchical predictive-coding principles provides a parsimonious explanation for the behavioral and neural findings. Together, these results reveal a pattern of large-scale information flow changes related to long-term priors’ involvement in visual perception, and provide constraints on future theories about the interactions between sensory processing and prior knowledge that underlie perception.

## Results

### Perceptual bias during bistable perception of ambiguous images

Fourteen participants implanted with standard clinical ECoG electrodes (grids and strips with 1-cm center-to-center spacing), including one participant additionally implanted with a high-density experimental grid (8 × 16 electrodes with 3-mm center-to-center spacing; see Fig. 1C for electrode locations pooled across all participants, and Supplementary Fig. 1 for coverage in individual participants), performed a bistable visual perception task in which they viewed ambiguous images and continuously indicated what they perceived (Fig. 1A). The images were the Necker cube and Rubin face-vase illusion, which induce perceptual switching between two possible interpretations of an image (hereafter referred to as percepts). Participants were asked to passively view the images (i.e. not volitionally hold onto a percept or intentionally switch between percepts), and report each time their percept changed using one of two buttons. They could also report if they perceived both or neither interpretation (‘unsure’). The mapping between the response buttons and the two percepts was alternated between blocks to dissociate perceptual content-related activity from movement-related activity.

Previous work has indicated that participants are often biased towards one of the two percepts, in a manner that reflects prior experience^25,27^. To test whether there is a perceptual bias at the group level, we analyzed the total percentage of time that each percept was experienced (Fig. 1B). For the face-vase image, there was a greater percentage of time spent perceiving the vase than the face although this difference was not significant (Wilcoxon sign-rank test, two-tailed: df = 13, signrank = 65, *p* = 0.46). For the Necker cube, participants spent significantly more time perceiving the green-fronted cube, which corresponds to a cube viewed from the top (df = 13, signrank = 89, *p* = 0.02).

For the Necker cube, the bias towards the view-from-above percept is consistent with previous reports^25,27^ and congruent with our frequent experiences of seeing cubes situated more often on the ground than above our head. For the Rubin face-vase image, any perceptual bias towards the vase may be related to the ‘object center’ bias^39^ due to the fixation cross being located on the vase portion of the image, or a ‘simplicity’ bias^40^ whereby participants are biased to interpret an image using a fewer number of objects (one vase vs. two faces).

We also calculated perceptual bias (*z*-value) at the individual-participant level (Supplementary Table 2) by comparing the set of durations of the two percepts (two-sided Wilcoxon sign-rank test). We shall hereafter refer to the percept that had longer durations (based on the sign of *z*-value) in an individual participant as that participant’s preferred percept, and the alternative percept as the (individual-specific) non-preferred percept. To examine whether individual perceptual bias was stable over time, we recorded 24 additional healthy participants performing the same task in three separate sessions, with adjacent sessions spaced >1 week apart. The reliability of individual perceptual bias across sessions was assessed using a one-way model intraclass correlation (ICC)^41^. Individual perceptual bias showed strong reliability for both images (FaceVase: ICC = 0.64, *F*_23,48_ = 6.38, *p* = 3.55e-8; Cube: ICC = 0.55, *F*_23,48_ = 4.67, *p* = 3.41e-6), suggesting that these biases are stable over multiple sessions spanning weeks, and supporting the idea that they, at least partly, reflect individual-specific long-term experiences. In addition, there was no significant difference in perceptual bias between the control group (averaged across three sessions, *N* = 24) and the ECoG patients (*N* = 14) (two-sided, Wilcoxon rank-sum test; FaceVase: *z* = 0.89, *p* = 0.37; Cube: *z* = 1.04, *p* = 0.30), suggesting that group-level perceptual bias is similar between patient and control populations. The sources of inter-individual variability in perceptual bias are beyond the scope of this study, but we speculate that factors such as an individual’s structural brain circuit^42,43^ and prior assumptions about the image^44^ could be important, both of which can be influenced by past experiences through plasticity or cognitive mechanisms, thereby contributing to long-term priors.

In what follows, we first localize neural activity underlying perceptual switching and perceptual maintenance, then describe directed neural influences across large-scale cortical networks that reflect individual-specific perceptual bias.

### Neural activity involved in perceptual maintenance and perceptual switching is spatially separate but shared between images

When viewing ambiguous images, why does our perception suddenly switch at times while at other times it seems so stable? Answering this question requires knowing which brain regions are involved in the switching and maintenance processes that are common for both preferred and non-preferred percepts. Previous fMRI studies have revealed that a network of frontoparietal regions exhibit enhanced activity during perceptual switching, although the functional role of such activity remains controversial^32^. Electrophysiological correlates of this prominent fMRI finding remain elusive^45–47^, partly due to the limited spatial resolution of scalp EEG.

**Fig. 2:**
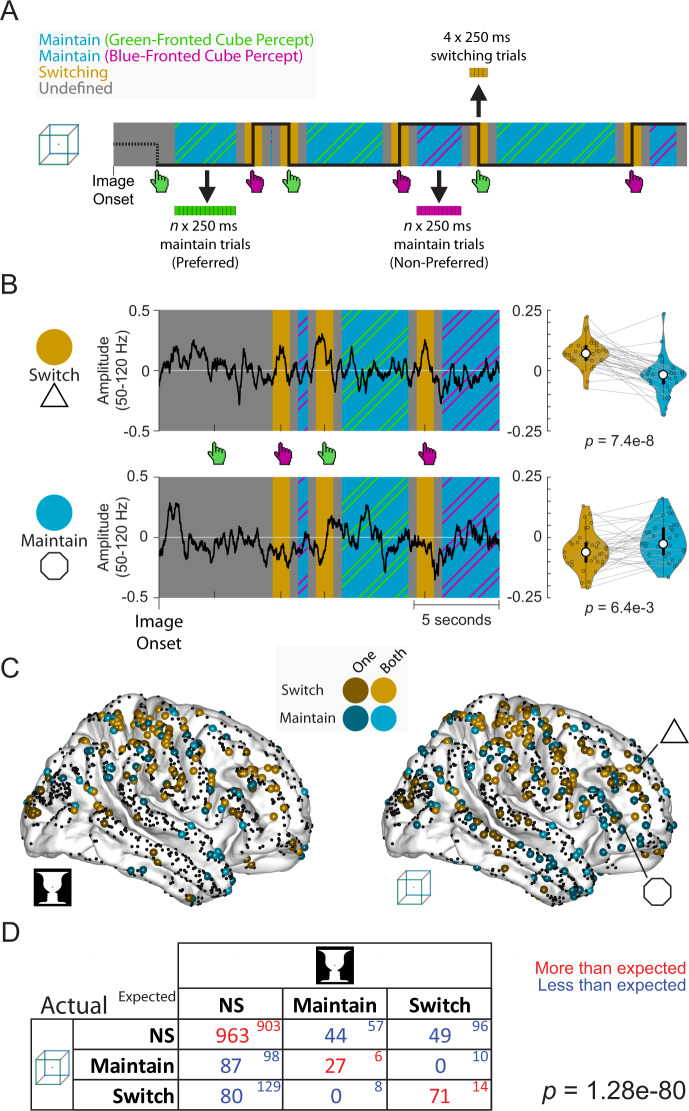
A common set of electrodes involved in perceptual switching and maintenance across two ambiguous images. **A** From each image presentation, time periods were extracted wherein the same percept is maintained (>1 s from button presses, ‘maintain’), and wherein the percept switched (<0.5 s from a button press, ‘switching’). For the Granger causality analysis in Figs. 3 and 4, these periods were split into 250 ms trials to improve data stationarity (see Methods section). **B** Left: example time courses of high-gamma amplitude (log-transformed, baseline-corrected; see Methods section) for two electrodes that show higher gamma activity during switching periods (top) or maintenance periods (bottom). Right: each switch period was paired with its subsequent maintenance period seen in the violin plot (thick circle indicates median, thick black line is the inter-quartile range, and thin black line extends to the most extreme data points not considered outliers); significance was assessed using a two-sided paired *t*-test. Electrode locations are marked in **C**. Data from participant 1 viewing the Necker cube image. **C** Locations of electrodes showing ‘switch’ and ‘maintain’ behavior. Lighter shades indicate electrodes with significant ‘switch’ or ‘maintain’ behavior for both ambiguous images; darker shades indicate electrodes with significant ‘switch’ or ‘maintain’ behavior for one image. **D** Joint distribution of the number of electrodes designated ‘switch’, ‘maintain’, or non-significant (NS) across cube and face-vase images (large font, ‘actual’), as well as the expected numbers of electrodes if category designation is independent between the two ambiguous images (small font, ‘expected’). A *χ*^2^ test was applied against the null hypothesis that the category of an electrode for one image was independent of its category for the other image, which was highly significant (*p* = 1.28e-80), suggesting that category designations have significant overlap between the two images. Source data are provided as a Source Data file.

To fill this gap, we first used the extensive intracranial electrode coverage in our dataset (*n* = 1321, *N* = 14; Fig. 1C; see Supplementary Fig. 1 for electrode coverage in individual patients) to identify neurophysiological underpinnings of perceptual switching and perceptual maintenance. To this end, we defined time periods of perceptual switching and perceptual maintenance (Fig. 2A), with the maintenance periods being >1 s away from a button press, and switching periods being within ±0.5 s of a button press. For each electrode we then compared the (log-transformed) amplitude of high-gamma (50–120 Hz) activity (correlate of population neuronal spiking^48–50^) during these periods, and designated electrodes as ‘switch’ (or ‘maintain’) if they had significantly higher (or lower) amplitude during perceptual switching than perceptual maintenance (one-sample *t*-test, two-tailed, *p* < 0.05; see Fig. 2B for two example electrodes).

The distribution of switch and maintain electrodes across the cortex is shown in Fig. 2C (see Supplementary Fig. 2 for medial and ventral views). Qualitatively, ‘switch’ electrodes clustered around motor areas (likely related to the button presses) and regions of the frontoparietal network^51^ including the middle frontal gyrus and dorsal parietal cortex. ‘Maintain’ electrodes clustered in regions that have previously been implicated in encoding perceptual content information during perception of ambiguous images including the temporal lobe and inferior frontal gyrus^33,52^. Overall, ‘switch’ electrodes were located more dorsally than ‘maintain’ electrodes, with the MNI Z-coordinates (describing ventral-dorsal location) significantly different between them (Mann–Whitney, two-tailed: face-vase: *z* = 2.68, *p* = 7.39e-3; cube: *z* = 5.17, *p* = 2.36e-7).

Previous fMRI studies typically made region-level inferences by pooling activation magnitudes or activity pattern information across voxels in a brain region. Here, capitalizing on the high spatiotemporal resolution of ECoG recordings, we asked whether there was a common set of switch (or maintain) electrodes across the two ambiguous images, or if instead these electrodes were specific for each image. To this end, we tallied the overlap of the different groups (switch, maintain, and not significant) of electrodes for the two images (Fig. 2D), and compared it to the expected numbers if the two sets were independent. Our analysis rejected the null hypothesis that the category of an electrode for the Rubin face-vase image is independent of its category for the Necker cube (*χ*^2^(4) = 378.41, *p* = 1.28e-80). Instead, we found a strong overlap for congruent categories of electrodes across images, with many more electrodes showing the same behavior across the two images than expected by chance (Fig. 2D, red), and no switch electrodes for one image that were maintain electrodes for the other image.

Thus, we found widely distributed networks of electrodes involved in perceptual switching and maintenance processes, with the former located more dorsally than the latter. Electrodes involved in perceptual switching and maintenance were shared between the two different ambiguous images, suggesting a canonical network mechanism regardless of the specific perceptual content.

### A backbone of feedforward activity flow during bistable visual perception

**Fig. 3:**
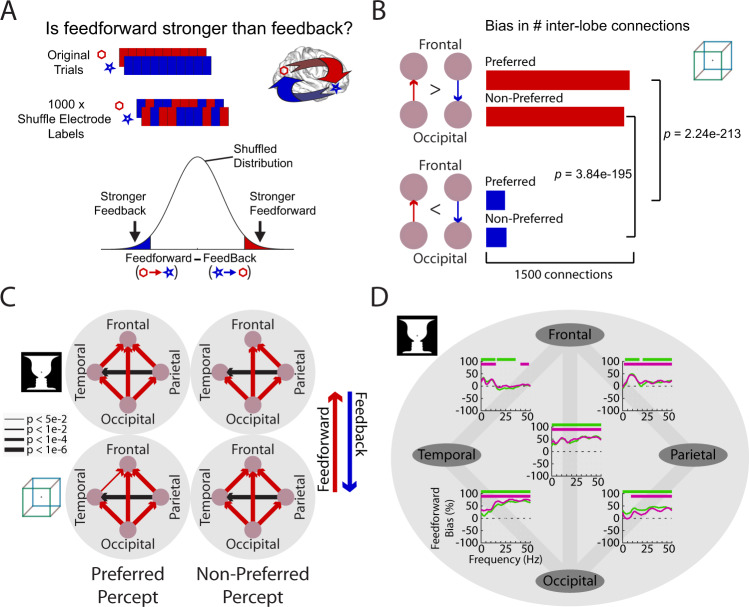
A backbone of feedforward activity flow during perceptual maintenance. **A** Granger causality was calculated separately for each direction between a pair of electrodes residing in different lobes. To assess significance, the difference in Granger causality between the two directions (‘asymmetry’) was compared with a null distribution created by shuffling the electrode labels 1000 times for each electrode pair. **B** To aggregate the results across many electrode pairs, a bias measure was calculated by comparing the number of significant inter-lobe connections in each direction using a two-sided binomial test, separately for each percept. **C** Significant (*p* < 0.05, uncorrected) biases (as assessed in **B**) in inter-lobe connections, separately assessed for 2 images × 2 percepts. Lobes were assigned a level in the cortical hierarchy (bottom: occipital; middle: temporal, parietal; top: frontal) and each directed inter-lobe connection between levels was defined as feedforward (red) or feedback (blue). Line width indicates the strength of significance. **D** Frequency-domain inter-lobe biases for the face-vase image during the preferred percept (green) and non-preferred percept (magenta). Positive and negative values correspond to feedforward and feedback biases, respectively. Horizontal bars: two-sided binomial test *p* < 0.05, cluster-corrected. Corresponding results for the cube images are shown in Supplementary Fig. 3. Source data are provided as a Source Data file.

To probe cortical information flow during bistable visual perception, we first characterized the overall information flow pattern during the perceptual maintenance periods (Fig. 2A, ‘maintain trials’). To this end, we calculated Granger causal influences—a measure of directed influences based on temporal precedence^53,54^—between simultaneously recorded electrodes in the same participant (see Supplementary Fig. 1 for electrode coverage in each patient, and Supplementary Table 3, top, for the number of electrode pairs analyzed). Previous macaque studies have shown that Granger causality applied to intracranial recordings can uncover visual hierarchy consistent with laminar projection patterns^55,56^ and cortical hierarchy involving the prefrontal cortex^57^. Here we focused on long-range inter-lobe connections and defined a large-scale three-layer cortical hierarchy including frontal, parietal/temporal, and occipital cortices (Fig. 3C), with occipital→parietal→frontal and occipital→temporal→frontal pathways corresponding to the dorsal and ventral visual streams, respectively^58^. This parcellation allowed us to pool electrode pairs across participants whose coverage varied by clinical needs (Supplementary Fig. 1).

We assessed the asymmetry between feedforward and feedback Granger causal influences for every electrode pair that resided in different lobes, and determined its significance by comparison to a null distribution (obtained by shuffling electrode labels, see Fig. 3A and Methods section). Thus, every inter-lobe electrode pair was designated as significantly biased in the feedforward or feedback direction, or not significant. We then assessed at the cortical lobe level whether there was a significant asymmetry in the communication between two lobes, by comparing the number of significantly biased connections in each direction using a binomial test (Fig. 3B). This analysis was performed separately using maintain trials for each of the four possible percepts (preferred and non-preferred percepts for the Rubin Face-Vase image and Necker Cube). The results suggest that, overall, feedforward influences outweigh feedback influences during this task—across both percepts of both images (Fig. 3C), consistent with the fact that our task involves visual perception driven by external sensory input.

We next assessed the contribution of different frequencies to the large-scale activity flow using a frequency-domain Granger causality analysis^59^. Feedforward input from occipital to temporoparietal cortices was primarily carried by high frequencies (>20 Hz) (Fig. 3D and Supplementary Fig. 3), consistent with previous intracranial findings in the visual hierarchy of the macaque^55,56^.

Together, this analysis reveals a backbone of predominantly feedforward activity flow during bistable visual perception. We next examined whether feedforward and feedback influences were modulated by the specific perceptual content experienced at a given moment and differ between the preferred and non-preferred percepts.

### Increased feedback influences during the preferred percept

Our main hypothesis suggests that long-term priors are recruited to guide perception of ambiguous images, resulting in an increased feedback drive when perceiving the preferred percept that is congruent with long-term prior, and an increased feedforward drive during the non-preferred percept which signifies a stronger prediction error. To test this hypothesis, we compared directed cortical influences between the two competing percepts for each ambiguous image.

**Fig. 4:**
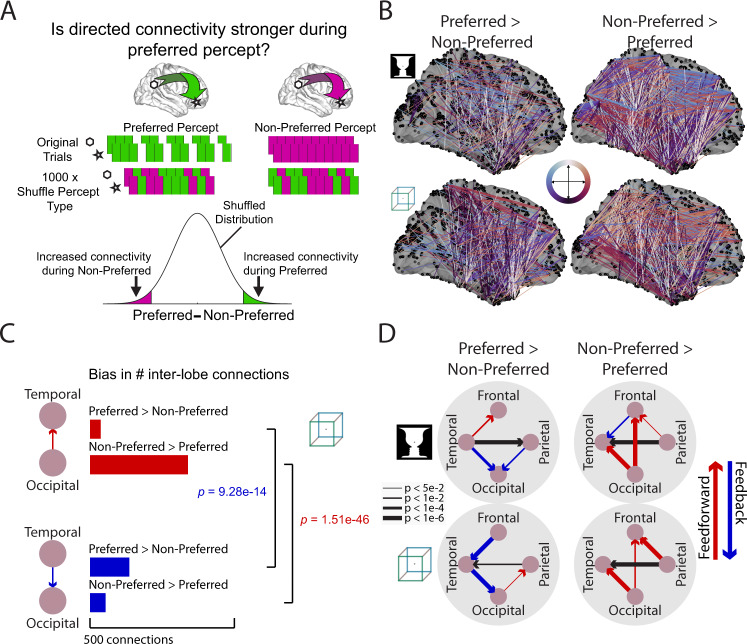
Increased feedback influences during the preferred percept and increased feedforward influences during the non-preferred percept across large-scale cortical networks. **A** For each inter-lobe electrode pair, Granger causality was calculated in each direction, separately for trials during preferred and non-preferred percept. Trials were dropped from the preferred percept to yield an equal number of trials between percepts. To assess significance, the difference in Granger causality between percepts was then compared to a null distribution created by shuffling the trial labels 1000 times. **B** Pairwise connections between lobes that showed significantly (*p* < 0.002, uncorrected) increased Granger causality during the preferred (left) or non-preferred (right) percept. Color indicates the direction of the connection on the Sagittal plane coded by the combination of anterior ↔ posterior and dorsal ↔ ventral directions (see center color wheel). Thus, feedforward connections are primarily red and feedback connections are primarily blue. Dorsal→ventral and ventral→dorsal connections are coded as black and white, respectively; medial-lateral dimension is not color-coded. **C** To aggregate the results across many electrode pairs, a bias measure was calculated by comparing the number of significant inter-lobe connections in each direction using a two-sided binomial test, separately for each perceptual preference. **D** Significant (*p* < 0.05, uncorrected) biases (as calculated in **C**) in inter-lobe connections for those that are stronger during the preferred percept (left) or stronger during the non-preferred percept (right). Line width indicates significance of binomial test. Source data are provided as a Source Data file.

To this end, we grouped ‘maintain’ trials (Fig. 2A) according to whether the participant perceived their preferred percept or their non-preferred percept (for individual-level perceptual bias, see Supplementary Table 2). For every (simultaneously recorded) electrode pair residing in different lobes (see Supplementary Table 3, top, for the number of electrode pairs analyzed), we calculated Granger causal influence in each direction (feedforward and feedback) for each percept. We then tested whether the causal influence (e.g., from electrode A to B) is significantly different between the preferred and non-preferred percept by comparing it with a null distribution (obtained by shuffling trial labels, see Fig. 4A and Methods section).

Consistent with our hypothesis, we found that the preferred percept is accompanied by an increased feedback drive and the non-preferred percept is accompanied by an increased feedforward drive: Fig. 4B plots significant (using an arbitrary high threshold of *p* < 0.002 for visualization, given the large number of significant connections) changes in causal influences between percepts, with connections that are stronger during the preferred (or non-preferred) percept shown to the left (or right). Connections are color coded by direction: posterior→anterior (approximating feedforward) are shown in red, and anterior→posterior (approximating feedback) shown in blue. It can be seen that the preferred percept is accompanied by increased feedback activity (more blue-purple colors) and the non-preferred percept is accompanied by increased feedforward activity (more red colors).

We quantified the pattern of information flow as follows: for each lobar pair in each direction, we calculated the number of connections (i.e., electrode pairs) that are significantly stronger during the preferred (or non-preferred) percept (for an example see Fig. 4C, colored bars). For each percept, we then assessed the asymmetry between the two directions (Fig. 4C, brackets). For instance, between temporal and occipital lobes, there are significantly more top-down connections that are stronger during the preferred percept (two-sided sign test, Cube: *z* = 7.45, *p* = 9e-14; FaceVase: *z* = 4.41, *p* = 1e-5), and significantly more bottom-up connections that are stronger during the non-preferred percept (two-sided sign test, Cube: *z* = −14.33, *p* = 1.5e-46; FaceVase: *z* = −16.44, *p* = 1e-60). The results for all inter-lobe connections are shown in Fig. 4D. The preferred percept elicits stronger feedback activity from the temporal cortex to occipital cortex for both images. The non-preferred percept elicits stronger feedforward influences from occipital cortex to temporal and frontal cortex and from parietal to frontal cortex. We observed inconsistent patterns in the temporal-frontal pathway between the two ambiguous images; a finding that we further discuss below.

For the above analysis we used all simultaneously recorded inter-lobe electrode pairs to increase statistical power. A control analysis including only connections where at least one electrode was involved in perceptual switching or perceptual maintenance (Fig. 2C; for electrode numbers per lobe see Supplementary Table 3, middle and bottom) revealed a similar pattern of changes in information flow (Supplementary Fig. 4B), with consistent feedforward information flow seen from occipital to temporal cortex during the non-preferred percept. We also examined whether the results held if we only included participants with significant perceptual bias (Supplementary Table 2). Again we observed a similar pattern of top-down and bottom-up information flow changes between the preferred and non-preferred percept (Supplementary Fig. 4A). A final control analysis investigated whether the results observed in Fig. 4D might be due to mismatched temporal distances to perceptual switching, since preferred percepts have longer durations. We selected sets of trials from the preferred and non-preferred percepts where the distribution of temporal distance from the nearest button press was matched (Supplementary Fig. 4C). Applying the same analysis approach, similar results were obtained (Supplementary Fig. 4D), suggesting that differences in information flow between the preferred and non-preferred percept are not due to a difference in the temporal distance to perceptual switching.

A previous meta-analysis of perceptual switching during bistable perception revealed a consistent set of involved regions across multiple fMRI and transcranial magnetic stimulation (TMS) studies^32^. To obtain a more fine-grained view of cortical information flow, we defined a set of seven regions of interest (ROIs) covering these regions (Supplementary Fig. 4E and Supplementary Table 4). Electrodes located within 20 mm of the ROI centers were assigned to each ROI. We then assessed information flow between these regions using the same method as was used for inter-lobe connectivity (Supplementary Fig. 4F). During the preferred percept there is an increased feedback drive from the middle frontal gyrus (MFG) to fusiform face area (FFA) for Necker cube. During the non-preferred percept there is an increased feedforward drive from occipital cortex to temporal-parietal junction (TPJ) for both images, and additionally from occipital cortex to FFA for the cube image. Overall these results are consistent with our main finding of increased feedforward drive during the non-preferred percept and increased feedback drive during the preferred percept.

We next assessed the contribution of different frequencies to these results using a frequency-domain Granger causality analysis. During the preferred percept there was increased feedback influences in low frequencies (<40 Hz) from temporal cortex to occipital cortex and from frontal to temporal cortex (~20 Hz); during the non-preferred percept there was increased feedforward influences from occipital to temporal cortex across a wide range of frequencies (Supplementary Fig. 5).

Together, these analyses reveal that directed influences across large-scale cortical networks are modulated by the specific perceptual content experienced at a given moment in a manner consistent with our main hypothesis: during the preferred percept, there is enhanced top-down input from the temporal to occipital cortices; during the non-preferred percept, by contrast, feedforward influences from occipital to temporal and prefrontal cortex are enhanced.

### A hierarchical predictive coding model of bistable perception

To shed light on the computational mechanisms underlying our findings, we constructed a computational model that integrates elements of attractor networks with hierarchical predictive coding. Previous theoretical work captured bistable perceptual switching using attractor-network models^60,61^. Perceptual switching in such models occurs due to three mechanisms: mutual inhibition, adaptation, and noise. Mutual inhibition is implemented by each population suppressing the other population. Adaptation prevents one population from being continuously dominant by gradually reducing its firing rate, in turn weakening the mutual inhibition and allowing the other population to take over. Finally, noise provides a second route to perceptual switching whereby random fluctuations in firing rate can drive alternations.

**Fig. 5:**
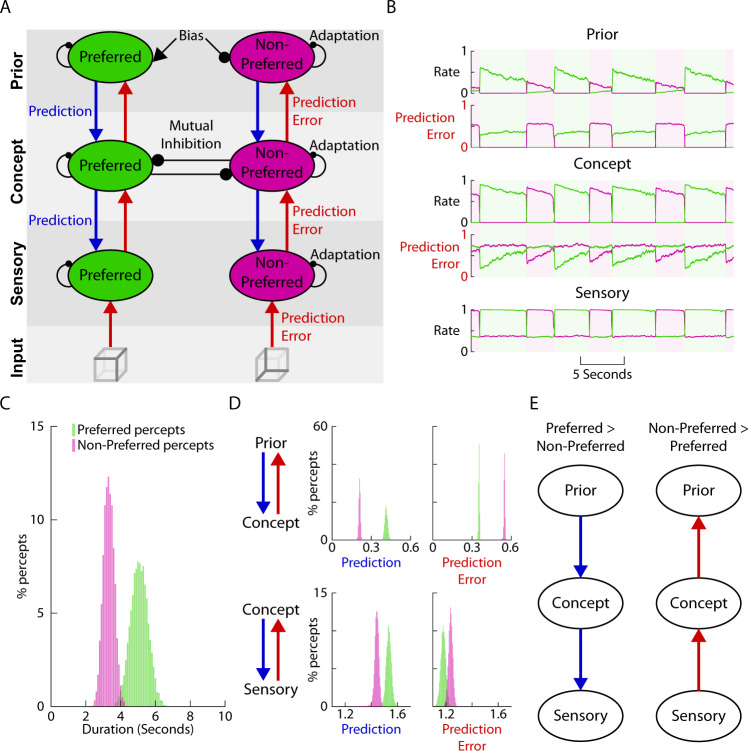
A computational model incorporating hierarchical predictive coding and attractor networks reproduces main experimental findings. **A** Model consists of three layers (sensory, concept, and prior), each containing neural populations whose activity is tuned to one or the other percept. Communication between layers is in the form of bottom-up prediction error and top-down prediction. Communication between populations tuned to different percepts only occurs within the concept layer in the form of mutual inhibition. Excitatory interaction is displayed as arrows, inhibitory interaction as circles. For model details see methods. **B** Example model output shows how rates of the populations tuned to the preferred (green) and non-preferred (purple) percept, respectively, change over time. The currently experienced percept is defined by the population with higher firing rate within the concept layer and is indicated by shading. Prediction-error inputs received by the prior and concept layers are also shown. **C** Preferred percepts have longer durations than non-preferred percepts. **D** The distribution of the strengths of prediction and prediction error signals between layers during the preferred (green) and non-preferred (purple) percept. **E** Summary of **D**: during preferred percept top-down prediction is stronger between layers, and during non-preferred percept bottom-up prediction error is stronger.

Previous attractor-network models, however, have not typically considered hierarchical interactions between brain regions (but see^62^). To understand the top-down and bottom-up interactions that ebb and flow according to perceptual content (Fig. 4), we first extended the classic attractor-network model to incorporate multiple layers. Motivated by multivariate decoding and connectivity patterns obtained from fMRI data during bistable perception, we previously proposed a two-layer architecture for bistable perception^33^, with populations tuned to each percept present within each layer, the lower layer representing sensory details, the higher layer representing concepts, and mutual inhibition only occurring within the concept layer. Building on this architecture, to incorporate a long-term prior, we added a third layer that introduces a bias term which continuously enhances the population representing the preferred percept and suppresses the population representing the non-preferred percept (Fig. 5A). Communication between layers is carried out by excitatory interactions between populations tuned to the same percept. As prediction and prediction errors have been strongly implicated in the mechanism of bistable perception^29,63^ we implemented a predictive coding form of communication between layers, whereby only unexplained activity from lower layers propagates up as prediction errors, and predictions—proportional to the activity in the higher layers—propagate down^29,64^. Model details are described in Methods sections, Computational Model.

The model exhibited the classic perceptual switching phenomenon, as seen in the alternation of firing rates between the preferred and non-preferred populations in all three layers (Fig. 5B). We defined the currently experienced percept based on the population with higher firing rate in the concept layer (green and purple shading in Fig. 5B), but since alternation was synchronized across all three layers, defining perceptual outcome based on the other two layers would yield similar results. The durations of the preferred percept were on average longer than those of the non-preferred percept (Fig. 5C), reproducing the behavioral finding of perceptual asymmetry (Fig. 1B).

We next investigated top-down and bottom-up activity flow in this network. To quantify top-down inputs, we summed the prediction signals across the two populations with different tuning preferences (Fig. 5A, green and purple), because our empirical analysis did not distinguish between them. Top-down prediction signals were stronger during the preferred percept compared to the non-preferred percept, both from the prior to concept layer and from the concept to sensory layer (Fig. 5D, left). This result reproduces the empirical finding that top-down inputs are stronger during the preferred percept from the temporal to occipital cortex (Fig. 4D). In the model, this pattern resulted from the preferred population firing at a higher rate during the preferred percept compared to the non-preferred populations’ firing during the non-preferred percept in the concept and prior layers (compare the ‘up state’ of the green and purple traces in Fig. 5B). This is in turn caused by the bias term that continuously increases the firing rate of the preferred population in the prior layer.

To quantify bottom-up inputs, we again summed the prediction error signals across the two populations with different tuning preferences. Bottom-up prediction error signals were stronger during the non-preferred percept compared to the preferred percept, both from sensory to concept layer and from concept to prior layer (Fig. 5D, right). This result reproduces the empirical finding that bottom-up inputs are stronger during the non-preferred percept (Fig. 4D). The stronger prediction errors occur due to the reduced firing of the population representing the non-preferred percept in the concept and prior layers when they are active (as compared to the preferred population during preferred percept), leading to reduced top-down prediction in this pathway and increased residual errors.

Together, this model explains how a static perceptual bias in the top layer can lead to cascading differences in communication between layers (Fig. 5E; see Supplementary Fig. 6 for the time courses of top-down and bottom-up signals during the evolution of a preferred or non-preferred percept): an increase in firing rate in the upper layer due to congruence with the prior bias leads to increased top-down flow and higher activity in the lower levels. A decrease in firing rate in the upper layer due to incongruence with the prior bias causes a cascade of lower activity in the lower layers due to reduced top-down predictions, which in turn leads to higher bottom-up prediction errors. Thus, we have shown that a relatively minimal set of mechanisms—including attractor network and hierarchical predictive coding—reproduces the empirical findings of perceptual asymmetry in the face of symmetric bottom-up evidence and the associated alternation between top-down and bottom-up influences across large-scale cortical networks.

## Discussion

In summary, we reveal large-scale cortical mechanisms underlying long-term priors’ role in guiding visual perception. Across two different ambiguous images, we observed that the preferred percept, which is congruent with long-term experiences, is accompanied by strengthened feedback influences. By contrast, the non-preferred percept is accompanied by increased feedforward influences. These results challenge theories proposing that long-term priors act predominantly in a bottom-up fashion^17,18^ and support theories suggesting that long-term priors reside in higher-order brain regions and act on perception through top-down feedback^20,21^.

Although it is well known that priors learnt through lifetime experiences powerfully shape perception—e.g., it is nearly impossible to see a shape with shading at the bottom as concave^16^—the neural mechanisms underlying long-term priors’ influence on perception remain poorly understood. To tackle this long-standing question, we collected an extensive ECoG dataset of bistable visual perception, with electrode coverage of all cortical lobes and a large number of simultaneously recorded electrodes in each individual participant, ideal for probing the dynamic information flow between cortical areas. Long-term experience’s influence on shaping perceptual asymmetry when viewing ambiguous images is well documented in the psychophysics literature^25,27^, but has been largely neglected in neuroscientific studies of bistable perception. This phenomenon allowed us to compare, under identical visual input, when perception is and is not congruent with long-term prior. Importantly, we presented two different ambiguous images to each participant, with the data from them separately analyzed, thereby providing a within-study reproducibility and generalizability check.

By investigating information flow patterns that differ between the two percepts for each ambiguous image, we observed that the preferred percept is accompanied by increased feedback influences from temporal to occipital cortex (Fig. 4C, D). This finding supports our hypothesis that prior knowledge learnt from lifetime experiences is recruited and fed back from higher-order brain areas to lower-order areas to guide perception of ambiguous sensory input. This finding challenges bottom-up views of long-term priors^14,17,18^, and is consistent with our computational model showing that by introducing a bias consistent with prior knowledge in the top layer, the model reproduced perceptual asymmetry and the cascading changes in neural activity (Fig. 5). By contrast, during the non-preferred percept, we found a robust increase in feedforward influences from the occipital to temporal and frontal cortices. This finding is consistent with our hypothesis motivated by the predictive processing framework^28–30^, suggesting that the non-preferred percept is accompanied by a prediction error signal manifesting as an increased feedforward drive in the same large-scale cortical network.

We constructed a simple computational model of bistable perception that combines the biophysical realism of attractor-network models including mutual inhibition, adaptation, and noise^60,61^ with hierarchy^33,62^ and predictive coding^29,63^. By incorporating mutual inhibition between neuronal assemblies at the same hierarchical level^33,60,61^ and a top-down bias consistent with prior knowledge learnt from past experiences, the model parsimoniously explains the asymmetry in perceptual competition observed behaviorally and the dramatic changes in large-scale cortical information flow associated with it. Our model is consistent with a previously proposed predictive-coding model of bistable perception^29^, but also provides a concrete computational implementation. Importantly, our experimental data provided the first direct neurophysiological evidence for the postulated top-down prediction and bottom-up prediction-error signals across the cortical hierarchy^29^, in which the present computational model is grounded.

A line of previous work investigated which brain areas are involved in perceptual switching and perceptual maintenance during bistable visual perception. These studies reported neural correlates of perceptual switching in frontoparietal networks^32^ and of perceptual maintenance in visual areas^38^. We probed neurophysiological activity associated with perceptual maintenance and perceptual switching using the extensive intracranial recordings. We found a common set of electrodes involved in perceptual switching across both ambiguous images that were located primarily in dorsal frontoparietal areas, and a different set of electrodes involved in perceptual maintenance that were located primarily in ventral frontoparietal and lateral temporal cortices (Fig. 2C). These results align with previous fMRI findings^32,33^ and provide the first comprehensive view of electrophysiological cortical activity involved in the maintenance and switching of percepts during bistable visual perception. We note that due to the participant reporting the perceptual switch, the switch-related activity identified herein contains motor and decision-related neural activity. While ‘replay’ conditions and no-report paradigms^65^ have been used to separate these confounding activities in the context of binocular rivalry-induced bistable perception, such experimental manipulations are currently not possible with static ambiguous images such as those investigated herein.

Our study thus presents both neural mechanisms specific to each perceptual content (large-scale cortical information flow that varies between preferred and non-preferred percept) and neural mechanisms common to the competing perceptual contents (neural activity associated with perceptual switching and perceptual maintenance). An important direction for future studies is to elucidate the relationship between these content-specific and non-content-specific neural mechanisms. This question could be approached by investigating the interactions between neuronal groups coding the preferred/non-preferred percept at each level of the cortical hierarchy and the neuronal groups promoting perceptual switching/maintenance (e.g., by using multiple high-density grids in the same participant). Such investigation can further test and refine the computational model put forth herein.

Previous studies have debated whether neural activity involved in perceptual switching during bistable perception embodies an attentional mechanism^32^. This remains an open question since many of the brain regions involved in perceptual switching reside in frontoparietal areas known to be involved in the control of attention. A bottom-up attentional account, however, cannot explain our main finding of changes in cortical information flow with preferred vs. non-preferred percept. If the preferred percept was experienced more due to bottom-up effects such as salience then it should attract more bottom-up attention, but instead the non-preferred percept is associated with stronger bottom-up influences. Second, increased top-down influences during the preferred percept and increased bottom-up influences during the non-preferred percept are observed in the occipitotemporal ventral visual pathway outside areas involved in the volitional control of top-down attention (Fig. 4D). By contrast, a hierarchical predictive coding account postulating top-down prediction signals and bottom-up prediction error signals effortlessly explains our findings, as shown by the computational model presented herein.

In this study, we investigate long-term priors—i.e., priors that are stably encoded in the brain, reflecting repeated past experiences or genetic influences. At the same time, it is also important to consider the potential contributions of stimulus characteristics and eye movements to the present findings. Both factors are known to influence perceptual asymmetry during bistable perception^66^. For instance, the visual field location of a presented stimulus can influence perceptual asymmetry: When the center of a Necker cube is shifted to the right or higher than the fixation location, the ‘view-from-above’ percept is enhanced, and vice versa^67^ (also see Supplementary Fig. 7); when the Rubin face-vase image is presented in left or right visual field (instead of center fixation), the face percept is enhanced (Supplementary Fig. 7). Both of these effects can be explained by the bottom-up sensory information coming from the fovea being stronger than that from the periphery. However, it is unlikely that stimulus characteristics explain our findings. First, the images were always presented at center fixation; thus, at least in the case of the Necker cube, sensory evidence supporting the two percepts is entirely symmetric (although the involved sensory/low-level neural populations may still be asymmetric). Second, all of our neural data analyses were grounded in perceptual biases defined at the individual participant level. Our behavioral data collected in a group of healthy participants (N=24) suggest that perceptual bias within an individual is stable across multiple sessions spanning weeks (see Results). In addition, an online behavioral study (N=46) established that perceptual bias within an individual is strongly correlated across stimulus conditions (Supplementary Note 1). Third, our online behavioral study also confirmed that the perceptual bias for the Necker cube is not driven by the color scheme chosen (Fig. 1A), as swapping the positions of the blue and green edges resulted in an identical ‘view-from-above’ perceptual bias (Supplementary Note 1). Lastly, if stimulus characteristics or low-level asymmetry in sensory encoding were the primary factor driving perceptual asymmetry in this experiment, we should expect the opposite pattern to the present findings, as the preferred percept should be associated with stronger sensory information, and in turn, stronger bottom-up signaling.

The potential effect of eye movements is related to the known influence of stimulus visual field location on perceptual asymmetry. In our experiment, participants were asked to fixate at the center of the screen throughout the experiment (Fig. 1A), but we did not have eye-tracking capabilities within the patient rooms to confirm this on a trial-by-trial basis. However, an eye-movement account of our results is highly unlikely: First, in the ROI-based information flow analysis (Supplementary Fig. 4E–F), there was no significant influence of frontal-eye-field (FEF, a region involved in the control of eye movements) on other regions. Second, if the change in perception were primarily driven by eye-movements, then both the preferred and non-preferred percepts should be associated with a similar pattern of large-scale information flow related to eye-movements. This would be very different from the push-pull pattern of bottom-up vs. top-down information flow we observed.

We observed substantial inter-participant variability in the overall perceptual bias. While the view-from-above prior is a well-established long-term prior, formed from a lifetime of visual experiences and informative about an individual’s psychiatric status^25^, it is possible that individual participants’ perception is influenced by additional short-term biasing effects such as priming, adaptation, or volitionally applying more attention to one percept than the other^66–70^. Nonetheless, several reasons suggest that long-term priors provide the most parsimonious explanation for the perceptual bias observed herein: first, the present paradigm, involving long image presentations (60 s) and inter-trial interval (7–13 sec), did not involve classic manipulations that induce priming or adaptation; second, participants were instructed to avoid volitionally directing attention to influence perceptual outcome; third, the group-level perceptual bias for the Necker cube is consistent with the effect well documented in the literature; fourth, the behavioral data from a cohort of healthy participants performing this task in multiple sessions spanning several weeks demonstrated that the perceptual bias is stable within an individual over time.

To focus on neural activity underlying perceptual content, we investigated cortical information flow during ‘maintain’ periods, defined as >1 s away from a button press. Because the actual perceptual switch precedes the button press by a variable amount of time (i.e., reaction time) that is unknown on any given trial, this approach ensures the veracity of the perceptual content during analyzed time periods and avoids switch-related activity (e.g., those related to decision-making and report). It is possible that these ‘maintain’ periods contain neural activity related to post-perceptual associations (e.g., semantic associations) but such associations are unlikely to account for changes in large-scale bottom-up and top-down information flow as observed here, as post-perceptual semantic processing is likely instantiated by associative activations within the semantic/default-mode network^71–73^. Furthermore, our control analysis (Supplementary Fig. 4C–D) showed that matching the temporal distance to button press between preferred and non-preferred percepts yielded the same findings, suggesting that the observed cortical information flow is largely stable during the ‘maintain’ periods, thereby providing a potential neural underpinning to the momentarily stable perceptual experience in these brief periods. Future investigation using time-resolved Granger causality analysis may facilitate bridging the current results focused on perceptual maintenance with previous reports focused on perceptual switching e.g., de Jong et al.^37^.

The present study opens several questions: First, we investigated inter-lobe communication following a three-layer hierarchy (occipital → temporal/parietal → frontal), which is a relatively coarse measure of information flow. While this method was necessary to get a tractable large-scale cortical view of information flow from a set of participants with heterogeneous electrode placement, future studies with denser electrode sampling (e.g., using the HD-grid employed in one of the patients herein) could shed light on more fine-grained information flow. Second, because the electrode coverage varies from participant to participant (a necessary constraint of intracranial investigation in humans), understanding the neural basis of interindividual variability in perceptual dynamics and perceptual bias would require a larger ECoG sample than the present study. Third, the level in the visual hierarchy at which the bias occurs merits additional investigation. While we show in the computational model that a bias (prior) at the top level of the hierarchy propagates down, it could also be the case that the bias occurs at a mid-level of the hierarchy and then propagates down to earlier levels. This could potentially explain why for the Necker Cube we observed feedback/feedforward changes consistent with a prior being located in frontal cortex, whereas for the FaceVase image, the feedback influences appeared to originated in temporal cortex. Fourth, a small proportion (28.6%, see Supplementary Table 2) of participants spent more time perceiving the blue-fronted cube, corresponding to a cube viewed from the bottom. This statistic is similar to that reported in a previous Necker cube study (reanalysis of data shows that 5 out of 16 healthy participants spent more time perceiving the view-from-below perspective)^25^. Currently, the source of this individual variability is unknown, although from an ecological perspective it is difficult to imagine that someone might have more experience viewing objects from below than from above. We speculate that an additional prior about whether the cube is floating or sitting could influence an individual’s perceptual bias and contribute to this individual variability. Such a mechanism would be similar to a recent observation that assumptions about background illumination influence an individual’s color perception in a highly robust and stable manner^74^. Lastly, studies have shown that long-term priors can be adaptively modified with training^75^. An interesting question for future investigation is how such training sculpts the large-scale cortical information flow that carries the effect of long-term priors, and whether training can restore perceptual priors absent in certain patient populations^25^.

Finally, we note that there is some evidence that patients with autism spectrum disorder exhibit aberrant bistable perceptual dynamics^76,77^ and an absent effect of the ‘view-from-above’ prior when viewing ambiguous images such as the Necker cube^25^ (although replications of these findings will be necessary to demonstrate their robustness^78^). Thus, the present findings may pave the way for a better understanding of the pathophysiology underlying perceptual disturbances in these patients^79^.

In conclusion, we demonstrate that long-term priors’ influence on perception is carried by top-down feedback inputs across the large-scale cortical hierarchy to occipital visual cortex. These top-down influences wax and wane with reciprocal bottom-up feedforward inputs in the same long-distance pathways that are consistent with prediction-error signaling^29,30^. These findings have implications for understanding how perception is shaped by lifelong experiences^20,21^, how the perceptual system resolves ambiguity that is pervasive in the natural environment^80,81^, and how perception might vary across individuals with or without neuropsychiatric illnesses depending on an intricate interplay between top-down and bottom-up processes^82,83^.

## Methods

### Participants

Fourteen epilepsy patients with implanted electrode strips and/or grids performed the bistable perception task while undergoing surgical evaluation with iEEG monitoring at NYU Langone Health Comprehensive Epilepsy Center. The experiment was approved by the NYU Langone Health Institutional review board and all patients provided written informed consent. All participants’ demographic and clinical characteristics are included in Supplementary Table 1.

To assess the within-participant stability of perceptual bias over time, we additionally recorded 24 healthy participants (mean age, 25.7; range: 19–37 yo; 15 females; all right-handed) performing the bistable perception task on three separate occasions, with adjacent sessions spaced at least 1 week apart. The study was approved by the New York University School of Medicine Institutional Review Board and all participants provided written informed consent.

### iEEG recordings

iEEG was recorded from implanted subdural platinum-iridium electrodes embedded in silastic sheets (2.3 mm diameter contacts, 10 mm center-to-center spacing, Ad-Tech Medical Instrument, Racine, WI). The decision to implant, placement of recording electrodes, and the duration of invasive monitoring were determined solely on clinical grounds and without reference to this study. Electrodes were arranged as grid arrays (8 × 8 contacts, 10 mm center-to-center spacing), linear strips (4 to 12 contacts) or some combination thereof.

One participant (#14) had an additional high-density grid (8 × 16 contacts, 1 mm diameter contacts, 3 mm center-center spacing, PMT corporation, Chanassen, MN) implanted over the occipital lobe. The participant provided written informed consent under the same IRB protocol with a specific question included about the implantation of a high-density grid. The occipital cortex is sampled less frequently in the typical ECoG patient population due to clinical needs. Data from this participant thus were crucial to boosting the statistical power to test our hypotheses regarding cortical information flow to/from the occipital visual cortex. The research grid implantation was carried out under an NIH BRAIN-funded project, which specifically targeted the visual cortex (NIH R01MH111417; PI: Devinsky).

Within 24 h after surgical implantation of electrodes, patients underwent a post-operative brain MRI to confirm subdural electrode placement. Electrodes were localized and mapped onto the pre-implant and post-implant MRI (or CT) using geometric models of the electrode strips/grids and the cortical surface^84^. The coordinates for each electrode were then transformed into the common MNI space. For this study, we automatically assigned each electrode to one of the cortical lobes (frontal, parietal, temporal, occipital) using the Brainnetome atlas^85^. We also defined ROIs using the coordinates used in^32^, and assigned electrodes located within 20 mm of the ROI centers to those ROIs.

### Clinical (macroelectrode) recording equipment

Recordings from iEEG electrode arrays were made using one of two amplifier types (as amplifiers were upgraded during the period of the study): NicoletOne amplifier (Natus Neurologics, Middleton, WI), bandpass filtered from 0.16–250 Hz and digitized at 512 Hz. The patient with the high-density grid (#14) was recorded with the Neuroworks Quantum Amplifier (Natus Biomedical, Appleton, WI) recorded at 2048 Hz, bandpass filtered at 0.01–682.67 Hz and then downsampled to 512 Hz. ECoG signals were referenced to a two-contact subdural strip facing toward the skull near the craniotomy site during the recording (and re-referenced offline during analysis to common-average reference). A similar two-contact strip screwed to the skull was used for the instrument ground.

### Experimental setup

Participants performed the task while sitting upright in their hospital bed with a laptop placed on a hospital table. Distance from the participant’s eyes to the center of the laptop screen was 55 cm, and all images presented subtended a visual angle of 12°. This choice of image presentation size was motivated by previous experiments using ambiguous images^67,86^ (see Supplementary Table 6). During the task the participants indicated their responses using the arrow keys on the laptop (← and → for the two percepts, ↑ for unsure). All participants gave their responses using their right hand. Triggers indicating task timing and button presses were sent via the laptop’s parallel port to the DC ports on the amplifier in order to synch task timing and ECoG data stream. The experiment was programmed in Presentation (Neurobehavioral Systems, Inc.).

### Task paradigm

The experiment consisted of blocks lasting approximately 7 min each, and was adapted from an experiment previously ran in fMRI^33^ using the same images. During each trial (Fig. 1A), participants first received an instruction screen which informed them which image was about to be presented (either Rubin’s Face-Vase or Necker Cube), and the response mapping for the three possible percepts (Vase/Face/Unsure or Green-fronted cube/Blue-fronted cube/Unsure). After a 2-second fixation period, the ambiguous image was then presented at the center of the screen for 60 seconds, while participants fixated on the cross in the center of the image. During this time participants pressed keys to indicate their current perception of the image (one key for each percept, and an additional key for ‘unsure’). Participants were instructed to press the ‘unsure’ key if they experience neither or both of the possible percepts. Six trials were presented during each block. Response mapping for the two percepts stayed constant throughout each block, but was alternated between blocks. The number of blocks recorded and analyzed for each participant are included in Supplementary Table 1. Across all 14 participants, two blocks were recorded but were not analyzed, one due to electrodes becoming disconnected due to patient movement, and one due to the participant dozing off during the block.

### Behavioral analysis

For each ambiguous image presentation (60 s), time periods were split between consecutive button presses. The time periods before the first button press and after the final button press during an image presentation were excluded from the analysis. The percentage of the total amount of time spent in each possible percept (Fig. 1B) were then calculated for each participant. Group-level perceptual bias effects were assessed by a Wilcoxon sign-rank test across participants for each ambiguous image separately. For individual participant-level preference, subsequent time periods of the different percepts (from the same image presentation) were paired, and a Wilcoxon sign-rank test applied (Supplementary Table 2).

### ECoG data pre-processing

ECoG data was imported into MATLAB using the Fieldtrip toolbox^87^, and then split into individual task blocks. The power spectrum and raw signal of each electrode were manually inspected. Noisy channels were removed. Sources of ‘noise’ excluded from analyses included saturation, muscle- and movement-related artifacts, epileptiform activity and poor contact. Data were then detrended and band-stop filtered to remove line noise and its harmonics (zero-phase-shift, 3^rd^-order Butterworth filter centered on 60, 120, 180, and 240 Hz, with 2–4 Hz bandwidth dependent on participant). On inspection of the power spectra it was observed that some channels had a strong peak at 1–2 Hz that did not appear to be neurophysiological in origin. Aligning this artifact to the electrocardiogram (ECG) showed that this oscillation was tightly coupled to the heartbeat, which could plausibly be caused by slight movement of the electrode due to blood vessel pulsation. To clean this artifact we adapted a previously published heartbeat removal algorithm^88^ (see *Heartbeat artifact removal*). After artifact cleaning, the data from each electrode were re-referenced to the common-average reference.

### Heartbeat artifact removal

For each participant that had an artifact-free ECG signal recorded and a heartbeat-related artifact present in the ECOG data (*N* = 10), an algorithm was applied to remove this heartbeat-aligned component without distorting the rest of the signal^88^. First, heartbeats were detected as threshold crossings of the ECG signal. Then for each ECoG electrode, the signal was split into a set of heartbeat-aligned trials which had the duration equal to twice the median of the inter-heartbeat interval, and were centered on the time of the heartbeat. The trial-averaged heartbeat-evoked waveform was then low-pass filtered (zero-phase-shift 3^rd^-order Butterworth filter at <5 Hz), with a tapered window applied (Tukey window, 10% cutoff). This provided a template of the artifact component that could then be removed from the ECoG signal, time-synched to each heartbeat, without a discontinuity arising between neighboring heartbeats. For those participants without a clean ECG signal (N = 4), electrodes with heartbeat-related artifacts were removed from analyses.

### Switch and maintain analysis

Each electrode was assessed for perceptual switching- and maintenance-related behavior using high-gamma activity during ‘switching’ and ‘maintain’ periods. To extract high-gamma activity, the pre-processed signal of each electrode in each block was filtered at 50–120 Hz using a zero-phase-shift 3^rd^-order Butterworth filter. Then the amplitude envelope was extracted by taking the absolute of the Hilbert transform. The amplitude envelope timeseries was then log transformed (base 10) into approximately normally distributed data, and the mean (of the log-transformed amplitude envelope) of each block was removed.

For each image presentation (60 sec long), data were split into perceptual switching periods and perceptual maintenance periods (Fig. 2A, orange and blue). Perceptual-switching periods were defined as periods around the button press (from 500 ms before to 500 ms after a button press). Perceptual-maintenance periods were defined from 1 s after a button press to 1 s before the next button press.

Each electrode was assessed for perceptual switching- and maintenance-related behavior using the ‘switching’ and ‘maintain’ periods defined above. To this end, each perceptual-switching period was paired with the subsequent perceptual-maintenance period. The mean log-transformed high-gamma amplitudes during these periods were then compared using a paired *t*-test (*p* < 0.05, two-tailed). Electrodes were assigned as ‘switch’ if it showed higher gamma-band amplitude during switching periods, and ‘maintain’ if its gamma-band amplitude was significantly higher during maintenance periods. To assess whether the same group of electrodes participated in perceptual switching (or maintenance) across the two ambiguous images (Fig. 2D), independence of the category designations of electrodes for the Face-Vase and Cube images was assessed using a chi-squared test, where a significant finding rejects the null hypothesis that they are independent.

### Assessing information flow during perceptual maintenance with granger causality

In order to assess information flow during the maintenance of a percept, parametric Granger causality analysis was applied to each pair of electrodes that resided in different lobes using the MVGC Toolbox^59^.

For each block, broadband data for each electrode was filtered at <50 Hz (zero-phase-shift 3rd-order Butterworth filter), and downsampled to 256 Hz. ‘Maintain trials’ were then extracted from perceptual maintenance periods for each perceptual content (e.g. Green-fronted cube or Blue-fronted cube) separately, by splitting each perceptual-maintenance period into 250 ms non-overlapping windows (Fig. 2A, green and magenta). Equal numbers of ‘Maintain trials’ between percepts were selected for each image by dropping equally spaced trials from the percept with longer durations. The low-pass filtering and trial length were chosen to improve stationarity of the data within each trial (i.e., 250 ms window), which is necessary for Granger causality analysis.

Granger causality was applied (in time and frequency domain) to ‘Maintain trials’ from each percept separately. The model order for Granger causality was selected by first obtaining the optimal model order using the full model (Bayesian Information Criteria), and then selecting the median model order pooled across all pairs of electrodes. The model order used was 14 (54.7 ms). For frequency-domain Granger causality, the frequency resolution was 0.5 Hz. Granger causal influences calculated in the time and frequency domains were entered into the following two analyses.

### Feedforward-feedback asymmetry granger causality analysis

To assess whether there was an asymmetry in feedforward-feedback drive (Fig. 3) for an individual connection (calculated by time-domain Granger causality), a null distribution of Granger causal influence values was created for each inter-lobe electrode pair (Fig. 3A) by shuffling the electrode labels (1000 permutations) before applying Granger Causality analysis. Asymmetry for each connection was calculated as the subtraction of the feedback Granger causal influence from the feedforward Granger causal influence. Connections with significant feedforward bias had asymmetry >97.5 percentile of the null distribution, and significant feedback bias if less than 2.5 percentile of the null distribution (which is equivalent to *p* < 0.05 in a two-sided test) (Fig. 3A).

To assess whether there was an asymmetry in feedforward-feedback drive between lobes (Fig. 3B, C), the number of significant feedforward connections between lobes A and B was compared to the number of significant feedback connections. A binomial test assessed whether the imbalance of information flow was significant (*p* < 0.05, MATLAB function *signtest*, approximate method).

To assess asymmetry in the frequency domain, results from frequency-domain Granger causality analysis were used. Asymmetries at the individual connection and inter-lobe level were calculated for each frequency bin using a similar approach as for the time-domain analysis described above. Significant clusters of frequencies were identified by the following procedure, which corrects for multiple comparisons through a nonparametric permutation-based approach (Fig. 3D and Supplementary Fig. 3). A cluster was defined as a contiguous set of frequencies where the inter-lobe bias was significant (*p* < 0.05) and the sign of the inter-lobe bias was the same. The cluster size (‘summary statistic’) was the absolute value of the sum of the statistic from each of the binomial tests in this set of frequencies. The maximum cluster size was calculated for each of the 1000 permutations, and clusters from the original data were assigned as significant if their size was larger than the 95^th^ percentile of the maximum cluster size from the shuffled data (corresponding to *p* < 0.05, two-tailed cluster-based permutation test). This method was applied separately for each inter-lobe interaction.

### Changes in information-flow patterns with perceptual content and perceptual bias

To assess whether the strength of an individual directed connection (calculated by time-domain Granger causality) changes significantly between the preferred and non- preferred percept (Fig. 4), a null distribution of Granger causal influence values was created (Fig. 4A) by shuffling the trial labels (preferred vs. non- preferred percept) (1000 permutations) before applying Granger Causality analysis. As with the previous analysis (Fig. 3), all inter-lobe electrode pairs were assessed in this analysis. For each directed connection, percept-related change was calculated as the subtraction of Granger causal influence during non-preferred percept from that during the preferred percept. Directed connections with significant preferred-percept bias had percept-related change greater than the 97.5^th^ percentile of the null distribution, and significant non-preferred percept bias if less than the 2.5^th^ percentile of the null distribution (which is equivalent to *p* < 0.05 in a two-sided test) (Fig. 4A). In the plotting in Fig. 4B, it is possible that Granger causal influences in both directions for an electrode pair have significant percept-related changes, in which case two separate lines were plotted connecting the same pair of electrodes.

To assess whether maintaining a specific percept increased more the feedforward or the feedback drive between two lobes, we compared the number of significantly biased connections for that percept in each direction (Fig. 4C). A binomial test assessed whether the imbalance of information flow was significant (*p* < 0.05, MATLAB function *signtest*, approximate method). In a control analysis, we restricted the analyzed connections to inter-lobe electrode pairs where at least one of the electrodes was classified as a ‘Switch’ or ‘Maintain’ electrode (Supplementary Table 3, Supplementary Fig. 4B). The rest of the analysis was the same as described above.

To assess percept-related changes in directed influences in the frequency domain (Supplementary Fig. 5), percept-related changes for individual directed connections and inter-lobe asymmetries were calculated for each individual frequency. Significant frequency-domain clusters were assessed using the same cluster-based permutation method as described in the section above.

### Computational model

The computational model (Fig. 5A) consists of 3 layers [prior (P), concept (C), sensory (S)], each containing one neural population for the preferred percept (PP) and one for the non-preferred percept (NPP). Each neural population (layer_group_) has a mean firing rate associated with it (e.g. *P*_*PP*_ is the firing rate associated with the preferred population of the prior layer). Firing rates change over time according to the inputs it receives, which include adaptation, prediction error, and noise and can include perceptual bias, mutual inhibition, and prediction depending on which layer the population is in. Adaptation, mutual inhibition and noise are implemented as in Huguet et al.^61^. For the population *x*, adaptation (*α*_*x*_) changes over time according to:

1 $${\tau }_{a}\frac{{{\mathrm{d}}}{a}_{x}}{{{\mathrm{d}}}t}=-{a}_{x}+{{\mathrm{F}}}(x)$$

where *x* is the firing rate of population *x*, and *F* is the input-output function described below. Mutual inhibition only occurs at the concept layer, and is proportional to the rate of the other population in that layer (*e.g*. −*βC*_*NPP*_ is the mutual inhibition that the preferred population receives from the non- preferred in the concept layer). Noise (*n*_*x*_) is implemented as a separate Ornstein-Uhlenbeck process^61^ for each population *x*.

2 $$\frac{{{\mathrm{d}}}{n}_{x}}{{{\mathrm{d}}}t}=-\frac{{n}_{x}}{{\tau }_{n}}+\sigma \sqrt{\frac{2}{{\tau }_{n}}}{{\mathrm{\xi}}} (t)$$

Where ξ (*t*)is a white noise process with mean of 0 and standard deviation of 1.

Prediction error coming into a layer was calculated as the subtraction of the rate of that layer from the rate of the layer below^64^, with a minimum prediction error set to zero so that prediction errors effects were always excitatory (e.g. max(0, *δ*_*C*_(*S*_*PP*_ − *C*_*PP*_)) is the prediction error that the preferred population of the concept layer receives from the sensory layer). Prediction coming into a layer was implemented proportional to the rate of the layer above (e.g. *η*_*P*_*P*_*PP*_ is the prediction that the concept layer preferred population receives from the prior layer). The final term is Bias which is a constant representing a lifelong prior that increases the firing rate of the population representing the preferred percept in the prior layer, and suppresses the population representing the non-preferred percept.

The differential equations governing the evolution of firing rates are:

Prior Layer (with inputs: -adaptation, ±bias, +prediction error, +noise)

3 $${\tau }_{P}\frac{{{{{{\mathrm{d}}}}}}{P}_{{{{PP}}}}}{{{{{{\mathrm{d}}}}}}t}=-{P}_{{{{PP}}}}+F(-\varnothing {a}_{{P}_{{{{PP}}}}}+{{{{{\mathrm{Bias}}}}}}+{{{{{\mathrm{max}}}}}}(0,{\delta }_{P}({C}_{{{{PP}}}}-{P}_{{{{PP}}}}))+{n}_{{P}_{{{{PP}}}}})$$

4 $${\tau }_{P}\frac{{{{{{\mathrm{d}}}}}}{P}_{{{{NPP}}}}}{{{{{{\mathrm{d}}}}}}t}=-{P}_{{{{NPP}}}}+{{{\mathrm{F}}}}(-\varnothing {a}_{{P}_{{{{NPP}}}}}-{{{{{\mathrm{Bias}}}}}}+{{{{{\mathrm{max}}}}}}(0,{\delta }_{P}({C}_{{{{NPP}}}}-{P}_{{{{NPP}}}}))+{n}_{{P}_{{{{NPP}}}}})$$

Concept layer (with inputs: -adaptation, -mutual inhibition, +prediction error, +prediction, +noise)

5 $${\tau }_{C}\frac{{{\mathrm{d}}}{C}_{ {{{PP}}}}}{{{\mathrm{d}}} {{{t}}}}=-{C}_{ {{{PP}}}}+{{{\mathrm{F}}}}(-\varnothing {a}_{{C}_{ {{{PP}}}}}-\beta {C}_{ {{{NPP}}}}+ {{{{{\mathrm{max}}}}}}(0,{\delta }_{C}({S}_{ {{{PP}}}}-{C}_{ {{{PP}}}}))+{\eta }_{P}{P}_{ {{{PP}}}}+{n}_{{C}_{ {{{PP}}}}})$$

6 $${\tau }_{C}\frac{{{{{{\mathrm{d}}}}}}{C}_{{{{NPP}}}}}{{{{{{\mathrm{d}}}}}}t}=-{C}_{{{{NPP}}}}+{{{\mathrm{F}}}}(-\varnothing {a}_{{C}_{{{{NPP}}}}}-\beta {C}_{{{{PP}}}}+{{{{{\mathrm{max}}}}}}(0,{\delta }_{C}({S}_{{{{NPP}}}}-{C}_{{{{NPP}}}}))+{\eta }_{P}{P}_{{{{NPP}}}}+{n}_{{C}_{ {{{NPP}}}}})$$

Sensory layer (with inputs: -adaptation, +prediction error, +prediction, +noise)

7 $${\tau }_{S}\frac{{{{{{\mathrm{d}}}}}}{S}_{{{{PP}}}}}{{{{{{\mathrm{d}}}}}}t}=-{S}_{{{{PP}}}}+{{{\mathrm{F}}}}(-\varnothing {a}_{{S}_{{{{PP}}}}}+{{{{{\mathrm{max}}}}}}(0,{\delta }_{S}({I}_{{{{PP}}}}-{S}_{{{{PP}}}}))+{\eta }_{C}{C}_{{{{PP}}}}+{n}_{{S}_{{{{PP}}}}})$$

8 $${\tau }_{S}\frac{{{{{{\mathrm{d}}}}}}{S}_{{{{NPP}}}}}{{{{{{\mathrm{d}}}}}}t}=-{S}_{{{{NPP}}}}+{{{\mathrm{F}}}}(-\varnothing {a}_{{S}_{{{{NPP}}}}}+{{{{{\mathrm{max}}}}}}(0,{\delta }_{S}({I}_{{{{NPP}}}}-{S}_{{{{NPP}}}}))+{\eta }_{C}{C}_{{{{NPP}}}}+{n}_{{S}_{{{{NPP}}}}})$$

The input-output function (F) was modeled as a sigmoid function^61^.

9 $${{\mathrm{F}}}(y)=\frac{1}{1+{e}^{\frac{\theta -y}{k}}}$$

with threshold *θ* = 0.2 and *k* = 0.1. All differential equations were integrated using the Euler-Maruyama method with time step 1ms, and the model was run for 5 × 10^7^ timesteps. All parameters used are given in Supplementary Table 5. In preliminary testing, we found that model behavior is robust to a range of parameters.

### Reporting summary

Further information on research design is available in the Nature Research Reporting Summary linked to this article.

## Supplementary information

Supplementary Information

Reporting summary

## Data Availability

Raw data from the online behavioral experiment is deposited to the figshare repository and can be downloaded at: 10.6084/m9.figshare.16716106. For behavioral and ECoG data collected from ECoG patients, source data are provided with this paper. An excel sheet provides source data for all main and Supplementary Figure. In addition, processed data and scripts to reproduce all figures are available at: https://github.com/BiyuHeLab/NatCommun_Hardstone2021. Trial-level behavioral data from the ECoG patients can be found in the source data for Table S2. Because of their confidential nature, raw ECoG data cannot be released to the public, but preprocessed data can be made available in de-identified form, upon reasonable request to the corresponding author. The Brainnetome atlas used in this study can be downloaded from https://atlas.brainnetome.org/download.html. Source data are provided with this paper.

## Source data

Source Data

## Code availability

We used publicly available open source software toolboxes and custom scripts written in MATLAB to analyze our data. Source code required to run all simulations, as well as processed datasets and scripts required to generate all figures presented here, are available at: https://github.com/BiyuHeLab/NatCommun_Hardstone2021.
