# Supplementary material for: Long-term priors influence visual perception through recruitment of long-range feedback
Source: Nat Commun. 2021 Nov 1;12:6288. doi: 10.1038/s41467-021-26544-w (PMC8560909; doi:10.1038/s41467-021-26544-w)
Supplement: Supplementary file 1 — Supplementary Information [file 41467_2021_26544_MOESM1_ESM.pdf]

Long-term Priors Influence Visual Perception through Recruitment of Long-range Feedback

Supplementary Information

Hardstone et al.

Including:

1 Supplementary Note

7 Supplementary Figures and

6 Supplementary Tables.

SUPPLEMENTARY NOTE 1

To address the question of how stimulus characteristics including size, color, and spatial location influence perceptual bias, we conducted an online behavioral task (using Gorilla.sc). We recruited subjects using Amazon Mturk, who had the Mturk masters qualification. We further selected participants who were using a computer with a monitor, and reported that they were not colorblind. Subjects signed an informed consent and were paid for a 30 minute task (including 10 minutes of resting time).

Before the task, we first ran a screen calibration where participants matched the size of an image of a credit card on screen to an actual credit card, and also reported their distance from the screen. This enabled us to present images at a specified visual angle. Each participant was assigned to one of three images: the same Necker cube ('ViewFromAboveGreen') and FaceVase image as used for ECoG participants, and a Necker cube image where the blue and green edges were swapped ('ViewFromAboveBlue'). In the task, Subjects were asked to always fixate on a cross in the center of the screen, and report their perception of the image presented in different conditions (see below) for 60 seconds each. For the cube images, participants reported the color of the cube face that was closest (as in the ECoG study).

To assess the effect of image size, the images were presented in the center of the screen at 3 different sizes (Cube: 4, 8, 12 degrees, FaceVase: 8, 12, 16 degrees). Different sizes were used for the two images, as during piloting we did not experience perceptual switches for the FaceVase image at 4 degrees. This is also consistent with prior literature, which has typically presented the FaceVase image at a larger visual degree (8–24°, see Supplementary Table 6) than the Necker cube (3–14°). To assess the effect of spatial location, images with a size of 8° were presented with a 5° offset from the central fixation point in 4 locations (left/right/up/down). Each participant completed 2 trials of each condition (7 conditions total: 3 Sizes presented at fixation and 4 peripheral locations), making a total of 14 image presentations.

Sixty participants completed the full task (22 female, mean age 37.7; range: 25-66, handedness: 3 left-handed, 1 ambidextrous, 56 right-handed), and 14 participants were removed from further analysis due to not following task instructions. The analysis reported below includes 16 participants for the FaceVase image, 12 participants for the 'ViewFromAboveGreen' Cube, and 18 participants for the 'ViewFromAboveBlue' Cube.

For each stimulus condition we calculated the percentage of time perceiving the ViewFromAbove or Vase percepts (excluding unsure time). For the Necker cube, we first combined data across the two participants groups who were shown different color versions of the cube image ('ViewFromAboveGreen' and 'ViewFromAboveBlue'). In all of the 7 stimulus conditions (3 image sizes at fixation and 4 peripheral locations), participants on average tended to perceive the 'view-from-above' percept more often (Fig. S7, left). This bias was significant for 3 out of 7 stimulus conditions following Bonferroni correction (Fig. S7, left; *: two-tailed Wilcoxon sign-rank tests, p<0.05, Bonferroni corrected). Importantly, there was no significant effect of image size ($F_{2,87}$=0.32, p=0.73) or image location ($F_{4,147}$=2.13, p=0.08) on perceptual bias.

To assess the effect of the coloring scheme of the cube image, we compared the perceptual bias for each of the 7 stimulus conditions between the two versions of cube image (ViewFromAboveGreen and ViewFromAboveBlue). There was no significant differences between the two versions of the cube image (two-tailed Wilcoxon rank sum tests; all p>0.05). A two-way 2x7 ANOVA with image color and presentation condition as the two factors also yielded no significant main or interaction effect (all p > 0.1).

For the FaceVase image, we found no significant group-level bias after Bonferroni correction across the 7 stimulus conditions (two-tailed Wilcoxon sign-rank tests), consistent with the lack of a

significant group-level effect in the ECoG patients. In addition, there was no significant effect of image size on perceptual bias ($F_{2,46}$=1.33, p=0.275), but there was a significant effect of spatial location ($F_{4,79}$=3.68, p=0.0086), where the vase percept was perceived more often in the up and down locations and the face percept was perceived more often in the left and right locations. This is likely because the faces are at the right and left flanks of the image and therefore would be closer to fovea when the entire image is presented in the left or right location.

To examine whether individual perceptual bias was stable across the 7 stimulus conditions (3 sizes, 4 locations), we assessed reliability using one-way model intraclass correlation (ICC) for participants who experienced perceptual switching throughout all the conditions (defined as having >10% of total time experiencing each percept). Individual perceptual biases showed significant reliability for both images (FaceVase: ICC=0.62, $F_{5,36}$=2.64, p=0.04; Cube: ICC=0.70, $F_{19,120}$=3.32, p=3.15e-5), supporting the idea that they—at least partly—reflect individual-specific context-independent experiences.

To summarize, we observed a strong group-level perceptual bias toward the 'view-from-above' percept for the cube image (same as for the ECoG participants; Fig. 1B), which was robust to the color, size, and visual field location of the image. There was a mild effect of visual field location consistent with prior studies (see Discussion), which did not reach statistical significance. For the face-vase image, we did not observe a significant group-level perceptual bias, consistent with results from the ECoG participants (Fig. 1B); in addition, there was no significant effect of image size. The spatial location effect for the face-vase can be explained by the asymmetry within the image itself. Lastly, an individual participant's perceptual bias is strongly correlated across different stimulus conditions. Together with behavioral results from a separate group of healthy participants (N=24, tested in the laboratory) showing that individual perceptual bias is stable across weeks (see Results, section "*Perceptual Bias during Bistable Perception of Ambiguous Images*"), these finding strengthen the conclusion that perceptual biases reflect individual-specific long-term priors.

SUPPLEMENTARY FIGURES

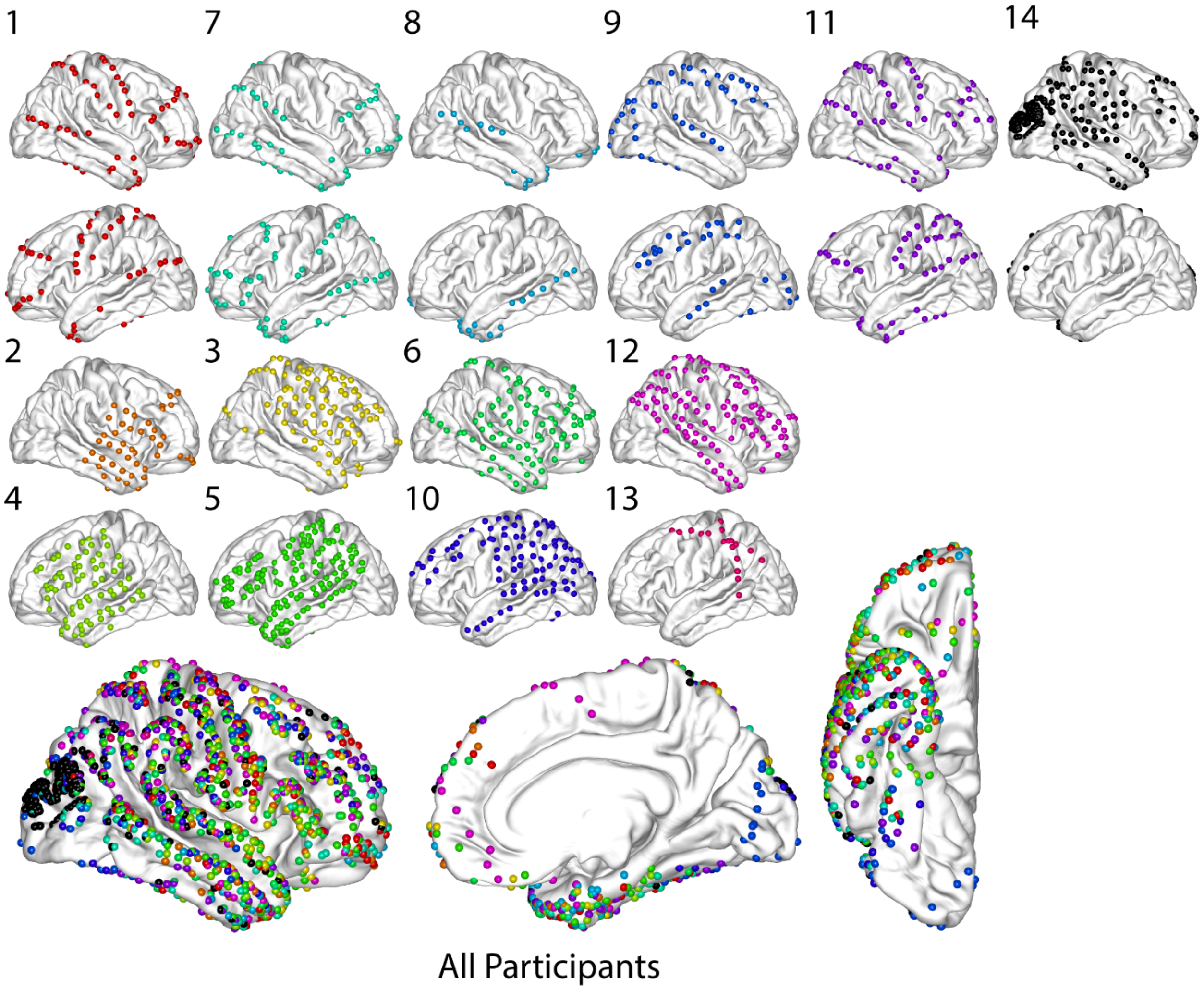

Supplementary Figure 1 (Complement to Fig. 1). Electrode localization. **Top**: Electrode locations for the fourteen participants implanted with regular clinical grids and strips, mapped onto MNI space. Subjects with bilateral coverage are shown in the top row. **Bottom**: electrode locations pooled across the 14 participants, colors indicate individual participants shown in the top; electrodes in the left hemisphere are displayed on the right hemisphere for visualization. Same as electrodes shown in black in Fig. 1C, but additionally showing the medial surface. Source data are provided as a Source Data file.

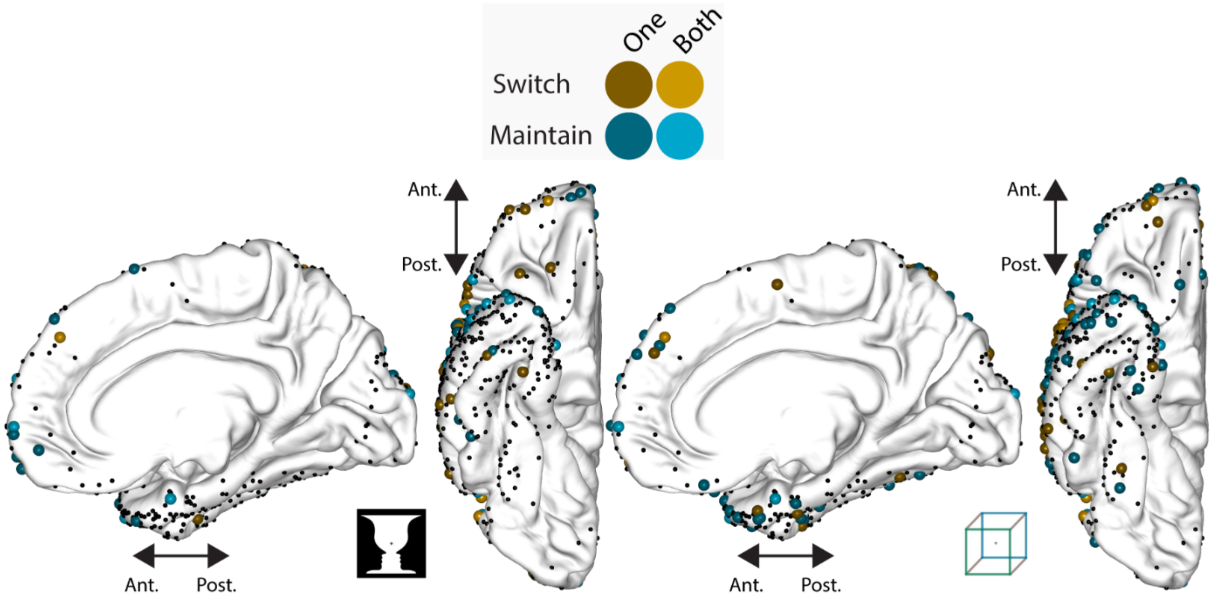

Supplementary Figure 2 (Complement to Fig. 2). Locations of electrodes showing ‘switch’ and ‘maintain’ behavior; same as Fig. 2C but displaying medial and ventral surfaces here. Lighter shades indicate electrode with significant ‘switch’ or ‘maintain’ behavior for both ambiguous images; darker shades indicate electrodes with significant ‘switch’ or ‘maintain’ behavior for one image. Source data are provided as a Source Data file.

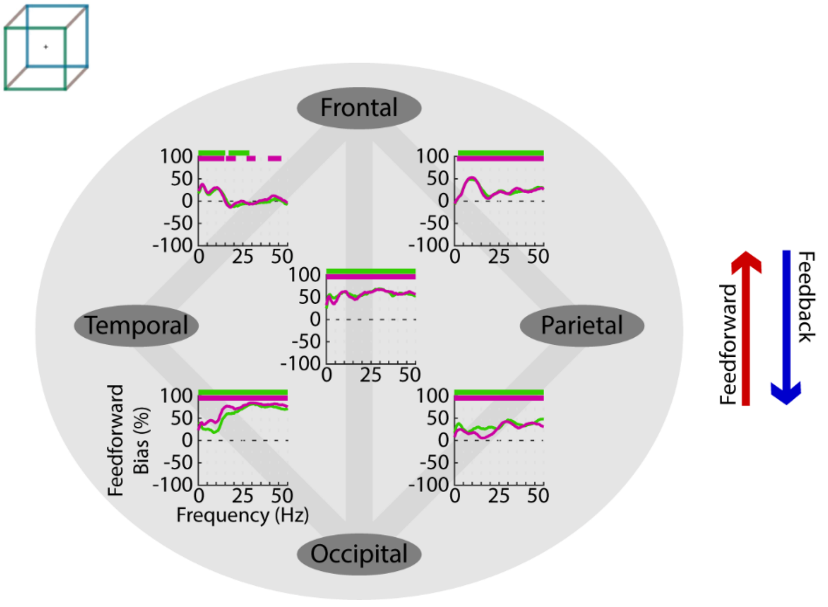

Supplementary Figure 3 (Complement to Fig. 3). Frequency-domain inter-lobe feedforward-feedback biases during the preferred percept (*green*) and non-preferred percept (*magenta*) of the Cube image. Horizontal bars: $p<0.05$, 2-sided binomial test, cluster-corrected. Format is the same as Fig. 3D. Source data are provided as a Source Data file.

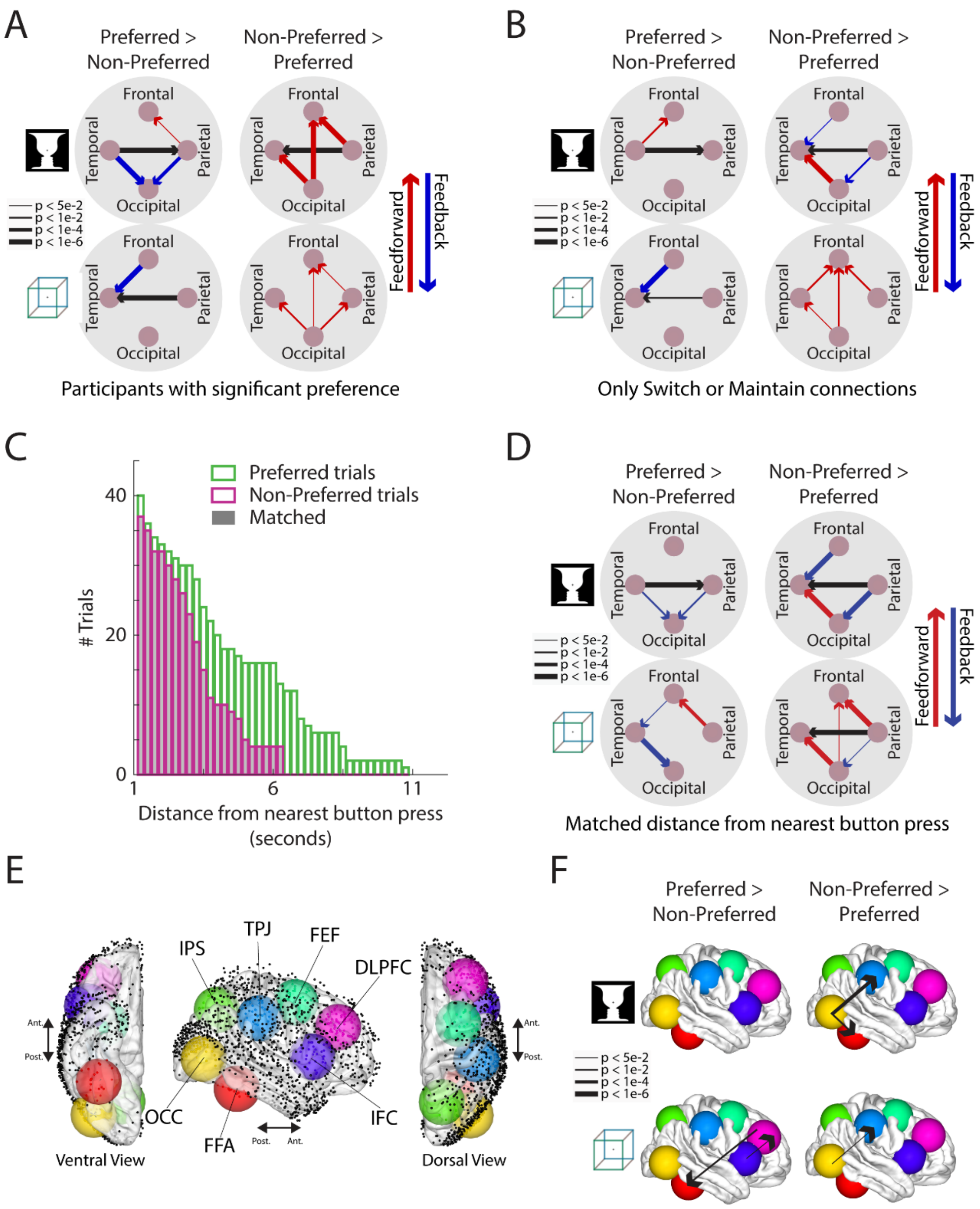

Supplementary Figure 4 (Complement to Fig. 4). (A) Same as Fig. 4D, except that only participants who had a significant perceptual bias (i.e., a significant preference for one of the two percepts, see Supplementary Table 2) were included in the analysis. Line-width indicates significance of 2-sided binomial test (uncorrected). **(B)** Same as Fig. 4D, except that only inter-lobe electrode pairs where at least one electrode exhibited significant 'switch' or 'maintain' behavior (Figure 2D) were included in the analysis. Line-width indicates significance of 2-sided binomial test (uncorrected). **(C)** The time distance of each 'trial' (i.e., 250-ms time window, see Methods) from the nearest button press was calculated, and overall distributions are shown for the preferred (green) and non-preferred (magenta) trials. Trials

were dropped from the preferred percept to match the distance distribution between the two percepts (gray). Example data from one participant are shown. **(D)** Same as Fig. 4D, except that trials for the preferred and non-preferred percept were selected so that their distributions of temporal distance to the nearest button press were matched within each participant. Line-width indicates significance of 2-sided binomial test (uncorrected). **(E)** Locations of 7 ROIs, colored spheres represent a 20-mm radius sphere around each ROI's center coordinate, which were used to identify ECoG electrodes within each ROI. **(F)** Same as Fig. 4D except that connectivity is defined between electrodes located within the 7 ROIs instead of lobes. Line-width indicates significance of 2-sided binomial test (uncorrected). Source data are provided as a Source Data file.

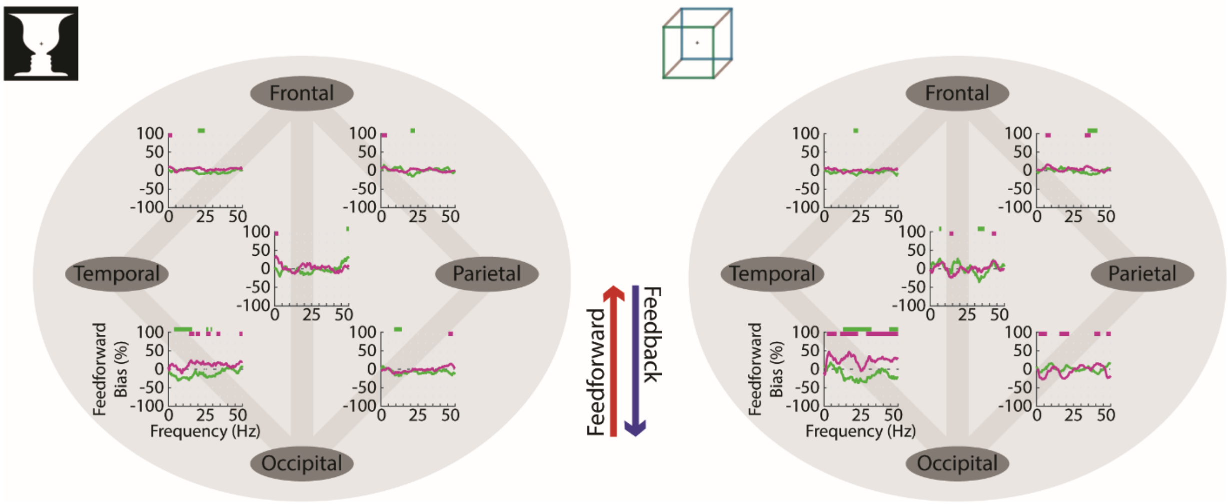

Supplementary Figure 5 (Complement to Fig. 4). (A) Frequency-domain Granger causality results corresponding to the time-domain analysis shown in Fig. 4D. Green and magenta lines show frequency-specific feedforward-feedback biases that are stronger during the preferred (*green*) or the non-preferred (*magenta*) percept, respectively. Feedforward biases are shown as positive values; feedback biases as negative values. Horizontal bars: $p<0.05$, 2-sided binomial test, cluster-corrected. Source data are provided as a Source Data file.

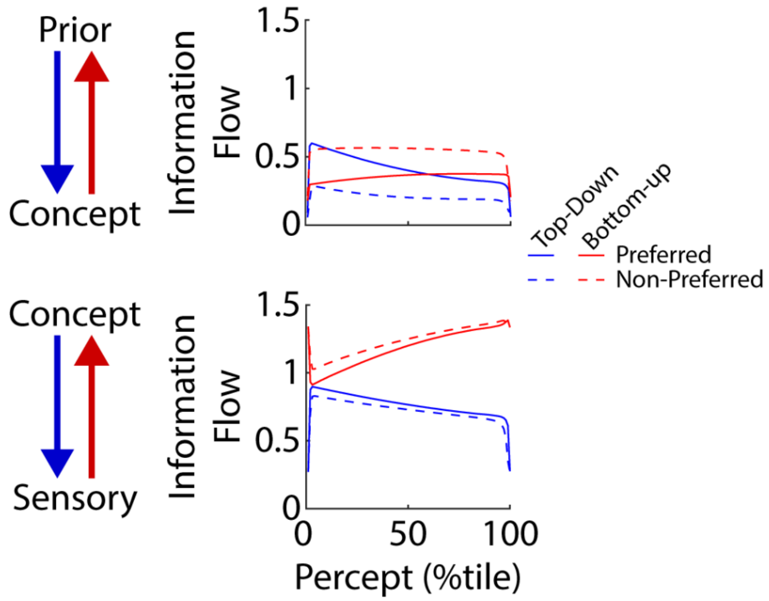

Supplementary Figure 6 (Complement to Fig. 5). Model prediction: top-down and bottom-up information flow between layers across the course of a percept. For each preferred and non-preferred percept, we calculated the total (i.e. summed across both populations) top-down and bottom-up information flow at 100 evenly spaced points across the percept duration.

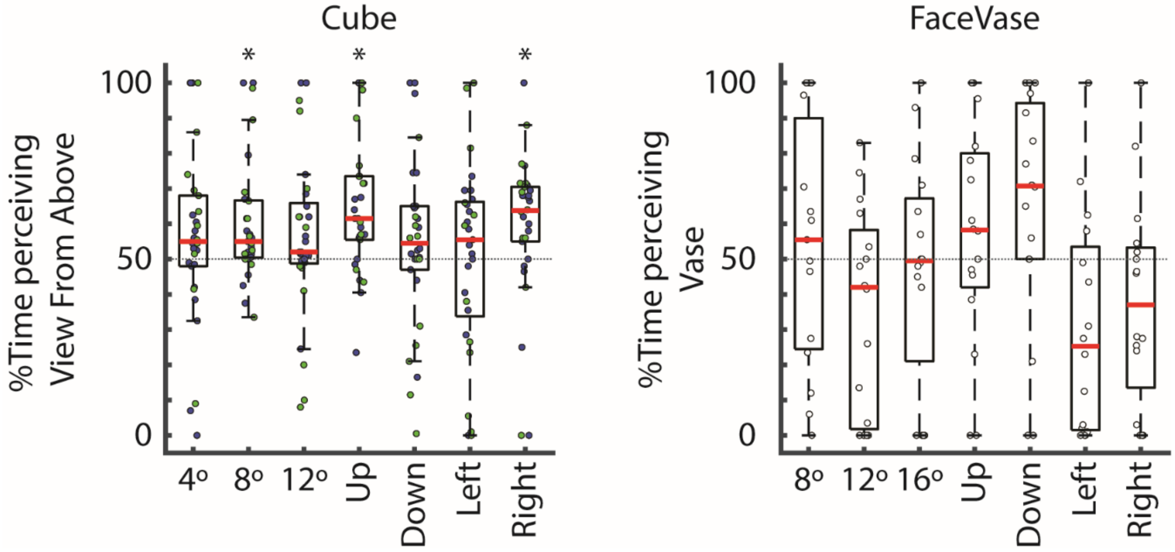

Supplementary Figure 7: Perceptual bias results from an online behavioral experiment (N = 60 total, 14 participants excluded for not following instructions). Dots represent individual participants; red lines indicate median across participants, the bottom and top edges of the box indicate the 25th and 75th percentiles, respectively. The whiskers extend to the most extreme data points not considered outliers. *Left*: (cube) green dots indicate participants presented with the ViewFromAboveGreen image (same as used in the main experiment, N = 12), and blue dots indicate participants presented with the ViewFromAboveBlue image (N = 18). *Right*: (FaceVase) N = 16. For each participant, perceptual bias was measured for stimuli presented at different sizes (4°, 8°, 12° for Cube; 8°, 12°, 16° for FaceVase) and different locations (image center offset 5° from center fixation, image size 8°). For detailed experimental procedures and statistics, see Supplementary Result. Source data are provided as a Source Data file.

SUPPLEMENTARY TABLES

Supplementary Table 1. Demographic, clinical and data collection information for each ECoG patient. Electrode localization can be found in Figure S1. Patients' age at surgery ranged from 18 to 44 year old (mean: 27.9; std = 8.1). Six (/eight) out of 14 patients are females (/males).

#	Handedness	Seizure Type	Seizure Focus	# used electrodes	#Blocks Recorded / Analyzed
1	Right	F2BTC	Bilateral multifocal	97	2/2
2	Right	FAS	Left temporal	56	2/2
3	Right	FIAS	Left temporal/insula	97	2/2
4	Right	FIAS	Left temporal	76	2/2
5	Left	FAS	Left anterior insula/temporal	144	2/2
6	Right	FAS/FIAS	Right temporal	104	2/2
7	Right	FIAS, F2BTC	Left and Right parietal/temporal	91	4/4
8	Right	FAS, FIAS, F2BTC	Right temporal	48	2/2
9	Right	FAS, FIAS	Left and Right temporal	78	2/2
10	Right	FAS, F2BTC	Left parietal/occipital	92	2/2
11	Left	FAS, FIAS	Right temporal	103	2/2
12	Right	FIAS, F2BTC	Right temporal/parietal /occipital	121	2/2
13	Right	FIAS, F2BTC, U	Left frontal	18	1/2
14	Right	FAS, FIAS, F2BTC	Right parietal	196	1/2

FAS=Focal aware seizure, FIAS=Focal with impaired awareness seizure, F2BTC= Focal to bilateral tonic-clonic seizure l, U=Unknown

Supplementary Table 2. Perceptual preference evaluated at the single-participant level. Percept durations for the two alternative percepts were compared using a Wilcoxon sign rank test (two-tailed), whereby consecutive percept durations for opposite percepts were paired, to control for any slow fluctuations in the data. Light gray shading (in the Z-value column indicates that participant preference is opposite to the group preference. Dark gray shading (in the p-value column) indicates significant preference at the individual-participant level ($p<0.05$).

	FaceVase			Cube		
**#**	**df**	**Z-value**	**p-value**	**df**	**Z-value**	**p-value**
**1**	17	-0.02	9.81e-1	30	2.03	4.28e-2
**2**	9	0.36	7.21e-1	8	0.42	6.74e-1
**3**	29	2.89	3.85e-3	36	2.59	9.54e-3
**4**	18	-0.28	7.78e-1	27	0.38	7.01e-1
**5**	39	1.90	5.81e-2	51	4.60	4.18e-6
**6**	22	2.54	1.11e-2	28	3.01	2.65e-3
**7**	88	1.06	2.88e-1	54	-0.75	4.51e-1
**8**	12	1.64	1.01e-1	16	1.45	1.48e-1
**9**	10	-0.27	7.90e-1	12	-0.47	6.38e-1
**10**	30	1.74	8.11e-2	47	1.87	6.11e-2
**11**	26	-1.06	2.90e-1	23	-0.21	8.31e-1
**12**	23	-0.40	6.89e-1	32	3.20	1.39e-3
**13**	3	0.37	7.15e-1	17	1.49	1.36e-1
**14**	6	-2.37	1.80e-2	7	-1.18	2.37e-1

Supplementary Table 3. Numbers of electrodes and electrode pairs analyzed across the 14 participants. Top (*All Electrodes*): the total number of useful/analyzed electrodes per lobe (right column), as well as the total number of inter-lobe electrode pairs (left-center table). Middle and bottom (*Switch/Maintain*): the total number of electrodes that showed significant 'switch' or 'maintain' behavior for each ambiguous image (right column), as well as the total number of inter-lobe electrode pairs where at least one electrode had significant 'switch' or 'maintain' behavior. For electrode coverage see Fig. S1.

	**#Potential Inter-Lobe pairs**	**Frontal**	**Temporal**	**Parietal**	**# Electrodes Per Lobe**
**All Electrodes**	**Frontal**				402
	**Temporal**	12678			413
	**Parietal**	12335	12087		401
	**Occipital**	2380	2654	6198	102
**Switch Maintain (FaceVase)**	**#Potential Inter-Lobe pairs**	**Frontal**	**Temporal**	**Parietal**	**#Switch/Maintain Electrodes per Lobe (FaceVase)**
	**Frontal**				69
	**Temporal**	2553			25
	**Parietal**	4001	3202		83
	**Occipital**	469	518	1800	14

Switch Maintain (Cube)	#Potential Inter-Lobe pairs	Frontal	Temporal	Parietal	# Switch/Maintain Electrodes per Lobe (Cube)
	Frontal				113
	Temporal	4539			52
	Parietal	5015	3432		89
	Occipital	872	460	826	11

Supplementary Table 4. ROI center coordinates.

ROI	x	y	z	# electrodes within 20mm
FFA	38	-49	-23	24
OCC	42	-77	3	60
IPS	18	-66	45	6
FEF	32	1	51	22
TPJ	48	-29	38	36
IFC	48	16	5	35
DLPFC	38	38	30	65

#Potential Inter-Lobe pairs	FFA	OCC	IPS	FEF	TPJ	IFC	DLPFC
FFA							
OCC	99						
IPS	16	13					
FEF	30	38	14				
TPJ	62	111	9	95			
IFC	34	40	12	11	102		
DLPFC	133	225	33	0	218	43	

Supplementary Table 5. Computational model parameters.

Description	Variable	Layer	Value
Rate Time Constants	τ_P, τ_C, τ_S	All Layers	10
Noise Time Constant	τ_n	All Layers	200
Noise Weight	σ	All Layers	0.02
Adaptation Time Constants	τ_a	All Layers	5000
Adaptation Weight	$\emptyset$	All Layers	0.8
Prior Bias (constant)	*Bias*	Prior	0.3
Prediction Error, Prediction Weights	δ_P, η_P	Prior	1
Mutual Inhibition	β	Concept	2
Prediction Error, Prediction Weights	$\delta_C, \delta_S, \eta_C$	Concept, Sensory	2
Input	I_{PP}, I_{NPP}	Sensory	0.8

Supplementary Table 6. Summary of the visual angle of the Rubin face-vase image and the Necker cube image employed in previous papers.

Paper	stimulus	Visual Angle	stimulus	Visual Angle
Wang, Arteaga, He PNAS 2013 [1]	Face-vase	19.4 x 14.2	Necker cube	15.9 x 13.2
Hesselmann…Kleinschmidt PNAS 2008 [2]	Face-Vase	14 x 14		
Parkkonen…Hari PNAS 2008 [3]	Face-Vase	24 x 24		
Meng Tong JoV 2004 [4]			Necker cube	8.2 x 8.2
Ozaki...Yamaguchi Cogn Neurodyn 2012 [5]			Necker cube	4.5 x 4.5
Britz…Michel Cereb Cortex 2009 [6]			Necker cube	2.5 x 2.5
Andrews...Blakemore Neuroimage 2002 [7]	Face-Vase	8 x 10		
Pitts...Hillyard Psychophysiology 2009 [8]			Necker cube	4 x 1.5
Kornmeier…Tebartz van Elst PLoS one 2017 [9]			Necker cube	5.5 x 6.5
Sato...Minami JoV 2020 [10]			Necker cube	7.7 x 7.7

SUPPLEMENTARY REFERENCES

1. Wang M, Arteaga D, He BJ. Brain mechanisms for simple perception and bistable perception. *Proc Natl Acad Sci U S A* **110**, E3340-E3349 (2013).
2. Hesselmann G, Kell CA, Eger E, Kleinschmidt A. Spontaneous local variations in ongoing neural activity bias perceptual decisions. *Proc Natl Acad Sci U S A* **105**, 10984-10989 (2008).
3. Parkkonen L, Andersson J, Hamalainen M, Hari R. Early visual brain areas reflect the percept of an ambiguous scene. *Proc Natl Acad Sci U S A* **105**, 20500-20504 (2008).
4. Meng M, Tong F. Can attention selectively bias bistable perception? Differences between binocular rivalry and ambiguous figures. *J Vis* **4**, 539-551 (2004).
5. Ozaki TJ*, et al.* Traveling EEG slow oscillation along the dorsal attention network initiates spontaneous perceptual switching. *Cogn Neurodyn* **6**, 185-198 (2012).
6. Britz J, Landis T, Michel CM. Right parietal brain activity precedes perceptual alternation of bistable stimuli. *Cereb Cortex* **19**, 55-65 (2009).
7. Andrews TJ, Schluppeck D, Homfray D, Matthews P, Blakemore C. Activity in the fusiform gyrus predicts conscious perception of Rubin's vase-face illusion. *Neuroimage* **17**, 890-901 (2002).
8. Pitts MA, Martinez A, Stalmaster C, Nerger JL, Hillyard SA. Neural generators of ERPs linked with Necker cube reversals. *Psychophysiology* **46**, 694-702 (2009).
9. Kornmeier J, Worner R, Riedel A, Tebartz van Elst L. A different view on the Necker cube-Differences in multistable perception dynamics between Asperger and non-Asperger observers. *PLoS One* **12**, e0189197 (2017).
10. Sato F, Laeng B, Nakauchi S, Minami T. Cueing the Necker cube: Pupil dilation reflects the viewing-from-above constraint in bistable perception. *J Vis* **20**, 7 (2020).
