# Supplementary material for: Long-term priors influence visual perception through recruitment of long-range feedback
Source: Nat Commun. 2021 Nov 1;12:6288. doi: 10.1038/s41467-021-26544-w (PMC8560909; doi:10.1038/s41467-021-26544-w)
Supplement: Supplementary file 2 — Reporting summary [file 41467_2021_26544_MOESM2_ESM.pdf]

nature research

Corresponding author(s): Biyu He

Last updated by author(s): Sep 15, 2021

Reporting Summary

Nature Research wishes to improve the reproducibility of the work that we publish. This form provides structure for consistency and transparency in reporting. For further information on Nature Research policies, see our Editorial Policies and the Editorial Policy Checklist.

Statistics

For all statistical analyses, confirm that the following items are present in the figure legend, table legend, main text, or Methods section.

n/a	Confirmed	
☐	☒	The exact sample size (n) for each experimental group/condition, given as a discrete number and unit of measurement
☐	☒	A statement on whether measurements were taken from distinct samples or whether the same sample was measured repeatedly
☐	☒	The statistical test(s) used AND whether they are one- or two-sided<br>*Only common tests should be described solely by name; describe more complex techniques in the Methods section.*
☐	☒	A description of all covariates tested
☐	☒	A description of any assumptions or corrections, such as tests of normality and adjustment for multiple comparisons
☐	☒	A full description of the statistical parameters including central tendency (e.g. means) or other basic estimates (e.g. regression coefficient) AND variation (e.g. standard deviation) or associated estimates of uncertainty (e.g. confidence intervals)
☐	☒	For null hypothesis testing, the test statistic (e.g. F, t, r) with confidence intervals, effect sizes, degrees of freedom and P value noted<br>*Give P values as exact values whenever suitable.*
☒	☐	For Bayesian analysis, information on the choice of priors and Markov chain Monte Carlo settings
☒	☐	For hierarchical and complex designs, identification of the appropriate level for tests and full reporting of outcomes
☒	☐	Estimates of effect sizes (e.g. Cohen's d, Pearson's r), indicating how they were calculated

Our web collection on statistics for biologists contains articles on many of the points above.

Software and code

Policy information about availability of computer code

Data collection	Behavioral data was collected using a custom experiment written in Neurobehavioural Systems: Presentation (v18.2). Concurrent neural data was collected using the clinical EEG system used by the epilepsy center.
Data analysis	We used publicly available open source software toolboxes and custom scripts written in MATLAB to analyze our data. Code supporting this study is available at https://github.com/BiyuHeLab/NatCommun_Hardstone2021.<br>Software and open source toolboxes used include:<br>MATLAB R2017a<br>MVGC toolbox (v1.0)<br>Fieldtrip toolbox (v20170509)

For manuscripts utilizing custom algorithms or software that are central to the research but not yet described in published literature, software must be made available to editors and reviewers. We strongly encourage code deposition in a community repository (e.g. GitHub). See the Nature Research guidelines for submitting code & software for further information.

Data

Policy information about availability of data

All manuscripts must include a data availability statement. This statement should provide the following information, where applicable:

- Accession codes, unique identifiers, or web links for publicly available datasets
- A list of figures that have associated raw data
- A description of any restrictions on data availability

Raw data from the online behavioral experiment is deposited to the figshare repository and can be downloaded at: https://doi.org/10.6084/m9.figshare.16716106. For behavioral and ECoG data col-lected from ECoG patients, source data are provided with this paper. An excel sheet provides source data for all main and

supplementary figures. In addition, processed data and scripts to re-produce all figures are available at: https://github.com/BiyuHeLab/NatCommun_Hardstone2021. Trial-level behavioral data from the ECoG patients can be found in the source data for Table S2. Be-cause of their confidential nature, raw ECoG data cannot be released to the public, but prepro-cessed data can be made available in de-identified form, upon reasonable request to the correspond-ing author. The Brainnetome atlas used in this study can be downloaded from https://atlas.brainnetome.org/download.html.

Field-specific reporting

Please select the one below that is the best fit for your research. If you are not sure, read the appropriate sections before making your selection.

- [ ] Life sciences
- [x] Behavioural & social sciences
- [ ] Ecological, evolutionary & environmental sciences

For a reference copy of the document with all sections, see nature.com/documents/nr-reporting-summary-flat.pdf

Behavioural & social sciences study design

All studies must disclose on these points even when the disclosure is negative.

Study description	Study including quantitative recordings of behavior and intracranial electrophysiological (ECoG) data.
Research sample	Research sample consisted of 14 patients who had ECoG strips and grids implanted while undergoing observation for epilepsy surgery. Demographic and clinical information pertinent to the interpretation of the results is provided in Supplementary Table 1 for all patients. Patients were selected to participate in this study based on relatively normal neuropsychology testing and a lack of confounding clinical factors such as diffuse brain abnormality. The sample is representative of patients evaluated at NYU Comprehensive Epilepsy Center as surgical candidates who are willing and able to participate in cognitive tasks.<br>In addition, 24 healthy volunteers participated in the longitudinal behavioral study in person, their aggregate demographic information is provided in the Methods section, under Participants. Sixty additional healthy volunteers participated in the online behavioral task; their aggregate demographic information is provided in Supplementary Note 1.
Sampling strategy	ECoG studies vary greatly in the number of patients studied. Previous ECoG studies of bistable visual perception have sometimes reported only a very small number of patients (e.g., N=2 in De Jong et al., Curr Biol 2020). Other cognitive studies have typically used numbers varying from 5 (e.g. He et al., PNAS 2008; Honey et al., Neuron 2012; Kucyi et al., J Neurosci 2018) to occasionally dozens (e.g., Manning et al., J Neurosci 2012). By using a relatively large patient sample, we were able to sample widely from different cortical regions covering all lobes, which enabled the present analysis of large-scale information flow.
Data collection	Subjects performed the task while sitting upright in their hospital bed with a laptop placed on a hospital table. Stimuli were presented on the laptop, and the distance from the subject's eyes to the center of the laptop screen was 55 cm, with all images presented subtended a visual angle of 12 degrees. During the task the subjects indicated their responses using the arrow keys on the laptop using their right hand. Triggers indicating task timing and button presses were sent via the laptop's parallel port to the DC ports on the amplifier in order to synch task timing and ECoG data stream. The experiment was programmed in Presentation (Neurobehavioral Systems, Inc.). The experimenter was present in the room throughout the task and was aware of the study conditions and hypothesis.
Timing	March 2016 - May 2019
Data exclusions	1 block from 2 subjects were excluded due to the patient falling asleep during the block (see Supplementary table 1)
Non-participation	No subjects dropped out
Randomization	Subjects were not allocated to experimental groups, since all subjects were presented with the same stimuli and task (within-subject paradigm). Stimuli were presented in a randomized order. Response mapping of first block was randomized across subjects.

Reporting for specific materials, systems and methods

We require information from authors about some types of materials, experimental systems and methods used in many studies. Here, indicate whether each material, system or method listed is relevant to your study. If you are not sure if a list item applies to your research, read the appropriate section before selecting a response.

Materials & experimental systems

n/a	Involved in the study	
☒	☐	Antibodies
☒	☐	Eukaryotic cell lines
☒	☐	Palaeontology and archaeology
☒	☐	Animals and other organisms
☐	☒	Human research participants
☒	☐	Clinical data
☒	☐	Dual use research of concern

Methods

n/a	Involved in the study	
☒	☐	ChIP-seq
☒	☐	Flow cytometry
☒	☐	MRI-based neuroimaging

Human research participants

Policy information about studies involving human research participants

Population characteristics	Patient characteristics represent the overall patient cohort at NYU Langone Medical Center with medically refractory epilepsy, and no conclusions were made in the manuscript about differences between patients characteristics (therefore there are no covariate-relevant population characteristics). Patients were 18-44 years old (mean 27.86, SD = 8.14), 6 patients were Female, 12 patients were right-handed (2 Left-handed).
Recruitment	Patients undergoing neurosurgery for the treatment of medically refractory epilepsy were given the opportunity to participate in research during their planned hospital stay. Patients who provided written informed consent were selected to participate in the present study based on relatively normal neuropsychology testing and a lack of confounding clinical factors such as diffuse brain abnormality. Only those who were up to performing perceptual/cognitive tasks during their hospital stay and had time availability participated in the study.
Ethics oversight	Study procedures were approved by the NYU Langone Health Institutional review board under protocol s14-02101 and all patients provided written informed consent.

Note that full information on the approval of the study protocol must also be provided in the manuscript.
